# PRTS: Predicting Single-Cell Spatial Transcriptomic Maps from Histological Images

**DOI:** 10.34133/research.0961

**Published:** 2025-11-06

**Authors:** Jingyi Wen, Lingxuan Zou, Jiying Liu, Xi Guo, Changkun Liu, Bensu Wang, Tongtong Deng, Chang Liu, Risheng Tang, Yanbin Yang, Yucheng Huang, Lijia Yang, Hui Wang, Zihao Li, Shengming Lin, Shipping Liu, Yuhu Zhang, Zhifeng Hao, Haiyu Zhou, Han Huang, Fei Ling

**Affiliations:** ^1^ School of Biology and Biological Engineering, South China University of Technology, Guangzhou 510640, China.; ^2^ School of Software Engineering, South China University of Technology, Guangzhou 510006, China.; ^3^ Department of Chemistry, University of Michigan, Ann Arbor, MI 48109, USA.; ^4^ Department of Neurology, Guangdong Neuroscience Institute, Guangdong Provincial People’s Hospital (Guangdong Academy of Medical Sciences), Southern Medical University, Guangzhou, Guangdong Province, China.; ^5^ School of Biomedical Sciences and Engineering, South China University of Technology, Guangzhou 511442, China.; ^6^ Department of Thoracic Surgery, Guangdong Provincial People’s Hospital (Guangdong Academy of Medical Sciences), Southern Medical University, Guangzhou 510080, China.; ^7^ BGI-Hangzhou, Hangzhou 310012, China.; ^8^ Department of Applied Health Science, The University of Illinois Chicago, Chicago, IL 60607, USA.; ^9^ Department of Mathematics, College of Science, Shantou University, Shantou 515063, China.; ^10^ Key Laboratory of Symbolic Computation and Knowledge Engineering of Ministry of Education, Jilin University, Changchun 130012, China.

## Abstract

High-resolution spatial transcriptomics (ST) data provide valuable insights into the molecular dynamics underlying complex biological processes. However, their widespread application remains limited due to high costs and technical challenges. Here, we present PRTS (Pathology-driven Reconstruction of Transcriptomic States), a novel framework that predicts single-cell-resolution ST data directly from histological images. Our results demonstrated that PRTS generated transcriptomic profiles for about 60,000 analyzable cell tiles per tissue section, representing an approximately 27-fold increase in analytical units compared to conventional ST spots and remarkably enhancing spatial resolution. Notably, PRTS achieves accurate cell-level transcriptomic predictions using only hematoxylin-and-eosin-stained tissue images. This method transforms costly ST technologies into a practical and scalable tool, offering a cost-efficient solution for comprehensive ST profiling in hematoxylin-and-eosin-based disease research.

## Introduction

The function of many biological systems, such as embryos [1], neural systems [2], and tumors [3], depends on the spatial organization of cells. Spatial transcriptomics (ST) technologies provide a powerful approach to studying spatial molecular changes in complex biological processes and related diseases. Existing ST technologies can be grouped into 2 approaches: in situ imaging-based and next-generation-sequencing-based technologies [4]. The former provides subcellular resolution but is limited by low gene throughput. In contrast, next-generation-sequencing-based ST technologies detect transcriptome-wide expression patterns but have a limited spatial resolution. Emerging methods like Stereo-seq [5] and Visium HD [6] offer genome-wide coverage with a subcellular resolution, overcoming the limitations of traditional technologies. This high-definition molecular atlas facilitates more accurate interpretation of cellular spatial organization, advancing in the understanding of tissue developments and disease mechanisms.

Despite the great potential of ST in medical research, its high cost and technical complexity currently limit its widespread application [7]. The limited availability of ST data makes them challenging to apply in clinical large-scale research and applications. Therefore, affordable and reliable methods are urgently needed for predicting spatial gene expression data in large-scale samples. Previous studies have shown that gene expression levels are correlated with histological image features. Several methodologies have been developed recently for ST data prediction with histological images. The Inferring Super-resolution Tissue ARchitecture (iStar) method was able to predict spatial gene expression with super-resolution from histological images [8]. Furthermore, the integrated graph and image deep learning (IGI-DL) method enabled the forecasting of the prognosis of cancer patients by predicting the gene expression patterns within specific regions on histological images [9]. However, these methodologies are mostly developed based on the data from Visium and Xenium, which limits their capacity to generate both high-resolution and comprehensive ST data. Moreover, they are unable to directly provide single-cell transcriptomics data.

To overcome these limitations, we present the deep-learning-based framework PRTS (Pathology-driven Reconstruction of Transcriptomic States), a method that predicts single-cell and spatially resolved transcriptomics from pathological images. Unlike previous approaches, PRTS is uniquely designed to predict the sparse, nucleus-focused transcriptome captured by platforms. This fundamental distinction necessitates a different technical implementation, as predicting a spot’s aggregate signal relies on a distinct set of histological components compared to predicting single-cell expression.

**Fig. 1.**
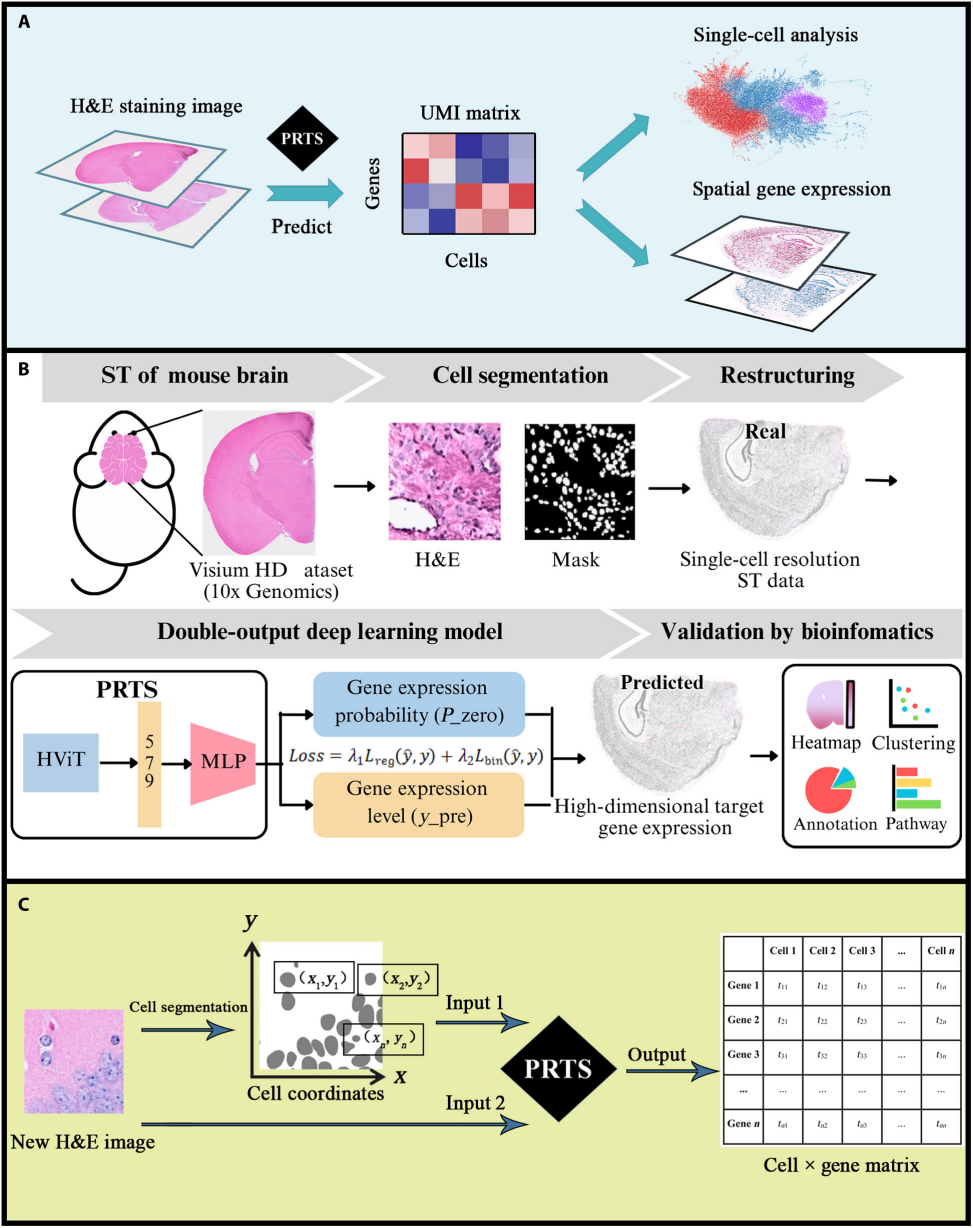
Overview of PRTS (Pathology-driven Reconstruction of Transcriptomic States). (A) The application of PRTS. PRTS bridges histological imaging and transcriptomics by predicting gene expression from hematoxylin and eosin (H&E) staining coronal sections. Based on coronal H&E images of the mouse brain, PRTS can predict the gene expression profiles of individual cells within the tissue. These predictions can be further utilized in downstream transcriptomic analyses, thereby bridging histological imaging, single-cell transcriptomics, and spatial transcriptomics. (B) General workflow and architecture of PRTS. (C) User input for PRTS. PRTS takes 2 inputs: a coronal H&E staining image and cell coordinates derived from cell segmentation of the same image (see Methods). The output is an .h5ad file containing a cell-by-gene expression matrix, which can be used for downstream analysis in Python packages such as Scanpy. UMI, unique molecular identifier; ST, spatial transcriptomics; MLP, multilayer perceptron.

PRTS is primarily trained using the publicly available Visium HD ST data and corresponding histological images from mouse brain coronal sections. The framework remarkedly predicts single-cell-level ST data (1,820 different genes) in similar types of histological images (Fig. 1A). Our method has established a more precise correlation between transcriptomes and economic histological images, which may greatly advance research on the organization of cells and their molecular basis.

As an expandable methodological framework, this study, although using the mouse brain coronal section as an example, enables researchers to apply our method to different Visium HD samples for training. We also leveraged cancer sections to explore the algorithm’s potential in pathological conditions. This flexibility aims to accelerate the large-scale application of spatial-related research across diverse biological scenarios.

## Results

### Training and evaluation process of PRTS

An overview of PRTS is shown in Fig. 1B. PRTS employs hierarchical histological image feature fusion architecture that aims to capture the fine-grained cell characteristics and microenvironment features of local cell populations. Due to the high sparsity of the expression matrix in a Visium HD dataset with a single-cell resolution, it is difficult to distinguish between zero-valued elements and low-valued elements. Thus, we designed a double-output neural network model that simultaneously determines whether a gene is expressed and predicts its expression level.

We used the publicly available Visium HD dataset of mouse brain coronal section as the training dataset. First, we performed cell segmentation on high-resolution hematoxylin-and-eosin-stained (H&E-stained) histological images (Fig. S1a to d) and obtained the gene expression patterns of each cell pixel region (Fig. S1e to g).

We performed quality control on the results of cell segmentation, and poorly segmented tiles were removed at this step to ensure the accuracy of cell tiles. Specifically, we excluded cell tiles with abnormally large nuclear areas, as well as segmented regions with very low nuclear unique molecular identifier (UMI) counts. The former likely represented aggregates of multiple cells, while the latter were likely nonnuclear regions. Such tiles accounted for only a very small fraction of the total (Fig. S1e and f). A total of 56,761 cells (Fig. S1h to l) and 1,820 highly variable genes (HVGs) were used for training (Table S1; for selection criteria, see Methods).

Through the above training paradigm, PRTS can recognize individual cells and predict the gene expression levels at their locations when new mouse brain coronal section H&E images are provided. The predicted results are presented in the form of a cell × gene expression matrix (Fig. 1C). Thus, PRTS not only meets the needs of ST analysis but is also compatible with popular single-cell RNA sequencing analyses, such as cell clustering, cell annotation, and functional enrichment analysis.

To assess the accuracy of PRTS in single-cell-resolved gene expression prediction, we applied it to another publicly available mouse brain section dataset from Visium HD. A total of 77,602 cell spots were used for validation. First, we numerically evaluated the performance of our model. The model achieved an accuracy of 81.39% in the binary-classification task of determining whether a gene is expressed (Table S2). Taking into account the various methods used to prepare the slices, this accuracy demonstrates PRTS’s consistency across multiple samples.

### PRTS-inferred expression patterns highly agree with actual tissue structures

**Fig. 2.**
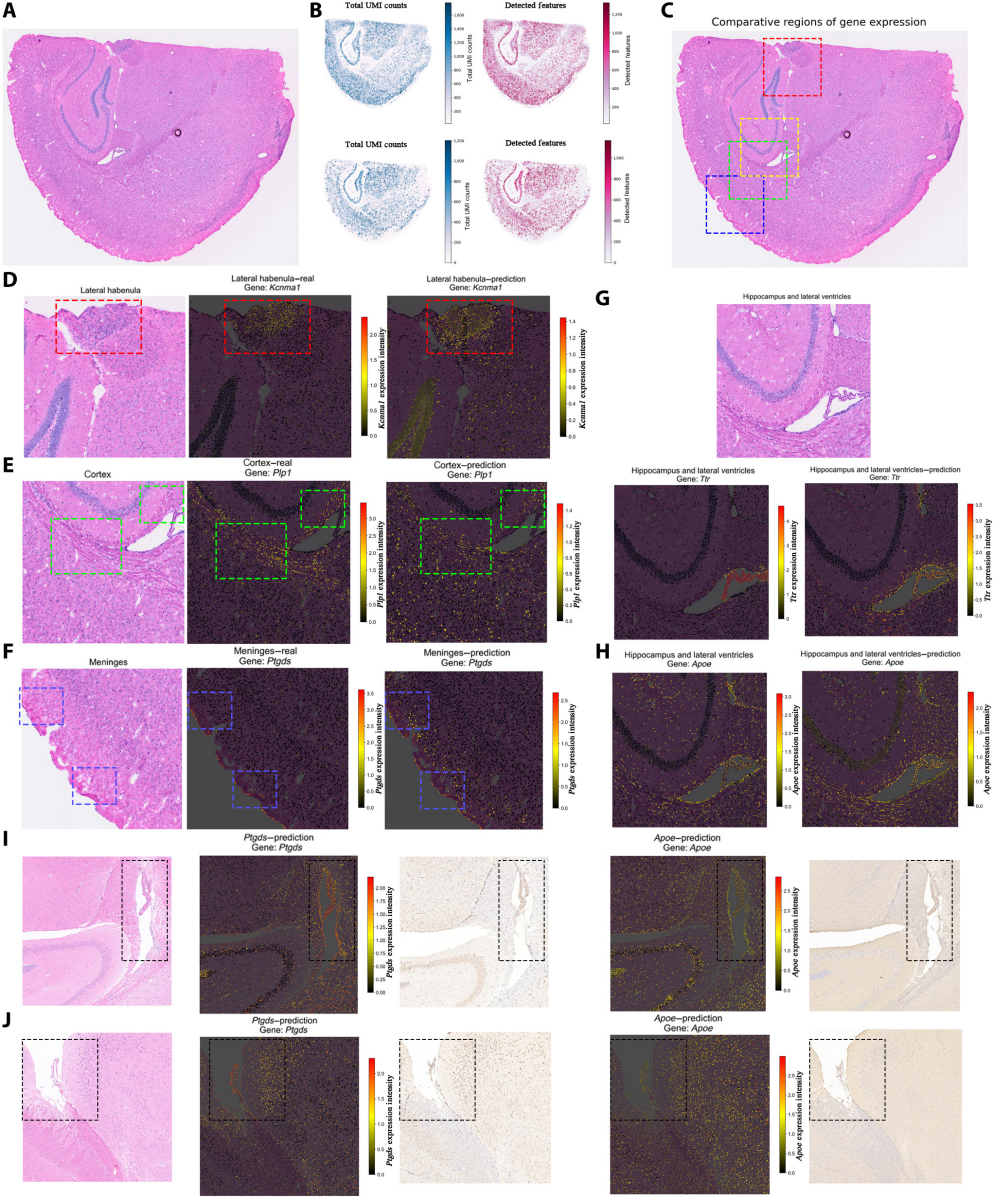
Performance of PRTS in predicting gene expression. (A) H&E staining image of the validation section, obtained from the publicly available Visium HD demo dataset (10x Genomics). (B) Prediction of total counts and number of features across the tissue. Top panels show ground truth from the dataset; bottom panels show model predictions. In the total counts map, each dot represents a cell, with darker blue indicating higher total counts. In the features map, darker red indicates a higher number of features. (C) Localization of selected subregions for gene visualization. Red box, *Kcnma1*; green, *Plp1*; blue, *Ptgds*; yellow, *Ttr* and *Apoe*. (D to H) Local regions used to compare predicted and ground truth gene expressions. For each gene expression map, color gradients from black to red indicate low to high expression levels. For panels (D) to (F): left, H&E staining image; middle, ground truth expression from Visium HD data; right, PRTS-predicted expression. Highlighted regions indicate cells with high expression levels. For panels (G) and (H), the same H&E image is shared in the top panel; bottom left, ground truth expression; bottom right, predicted expression. Expression of (D) *Kcnma1*, (E) *Plp1*, (F) *Ptgds*, (G) *Ttr*, and (H) *Apoe*. (I and J) Immunohistochemistry (IHC) validation of PRTS predictions. From left to right: H&E staining image, predicted *Ptgds* expression, Ptgds IHC result, predicted *Apoe* expression, and Apoe IHC result. (I) Lateral ventricle region; (J) pia mater region.

Firstly, we explored whether gene expression patterns (Table S1) are reliably predicted by PRTS. For the prediction of the global patterns, we evaluated the total counts and number of features for each cell. There are distinct variations in the total counts and number of features across various spatial locations, demonstrating the intrinsic biological complexity among different tissue structures [3,10–12]. In slices from the validation set (Fig. 2A), almost all cells in the hippocampus region exhibited higher total counts and feature numbers. In contrast, only scattered cells in the cortical and striatal regions showed a high level of expression, whereas cells in the meninges, corpus callosum, and ventricular–subventricular zone displayed relatively lower total counts and feature numbers. These distribution characteristics agrees with the ground truth measured by Visium HD (Fig. 2B).

We further focused on predicting key genes in neuroscience studies, including 4 genes with differential spatial and cellular expression (*Kcnma1*, *Plp1*, *Ptgds*, and *Ttr*), as well as the Alzheimer’s disease-related gene *Apoe* [13]. Virtually all PRTS-predicted expression distribution characteristics matched the ground truth. In general, the expression levels of specific genes are higher in regions enriched with their corresponding cell types (Fig. 2C to H). *Kcnma1* was enriched in neurons within the lateral habenula region (Fig. 2D and Fig. S3) [14]; *Plp1*, in oligodendrocytes within the subventricular zone and external capsule (Fig. 2E and Fig. S3) [15]; *Ptgds*, in the pia mater region (Fig. 2F and Fig. S3) [16]; and *Ttr*, in choroid plexus epithelial cells within the lateral ventricle region (Fig. 2G and Fig. S3) [17]. Notably, *Apoe* is widely expressed in the central nervous system, including astrocytes, microglia, vascular wall cells, and choroid plexus epithelial cells [18], and PRTS predicted higher *Apoe* expression levels in the ventricular–subventricular zone, lateral ventricle, and meningeal regions, where these cells are enriched (Fig. 2H and Fig. S3). This highlights our model’s generalizability across various cell types. We applied consecutively cut tissue sections to H&E staining and immunohistochemistry (IHC), and the expression features from IHC highly agreed with the predicted results with H&E images (Fig. 2I and J and Fig. S4). Moreover, we present the predicted expression patterns of 36 other key functional genes (Figs. S5 and S6 and Table S3) [19] to illustrate the broad predictive capabilities of PRTS.

Interestingly, the brain slices from the validation set were not fully sequenced. There is a small part on the left side of the slices that falls outside the spot-covered area (Fig. 2B). However, since PRTS only requires the histological images to generate the ST data, the predicted result has full coverage of complete slices, showing its capability of complementing the sequencing data under a real context.

### PRTS predicts single-cell ST data from histological images

**Fig. 3.**
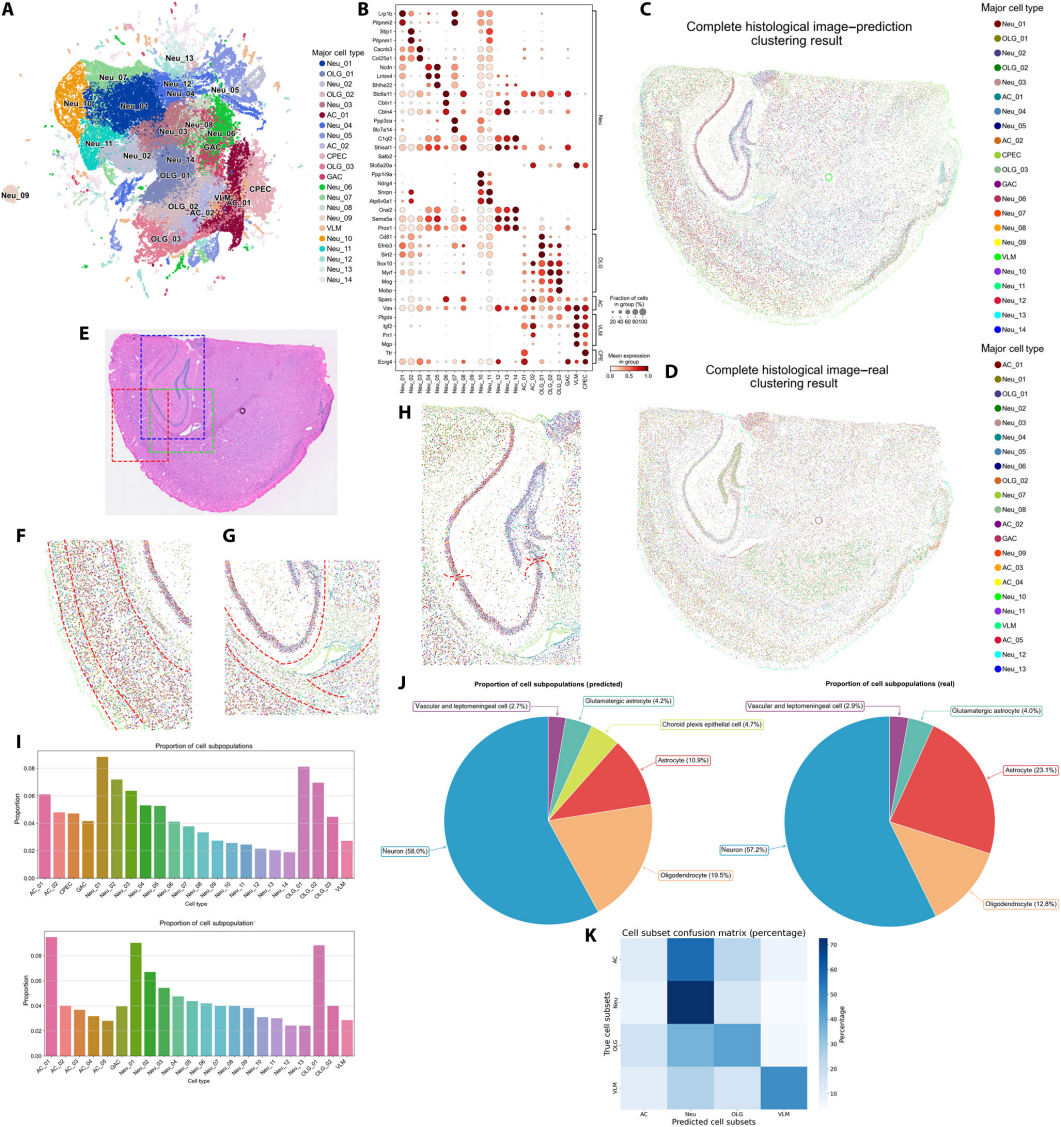
Single-cell RNA sequencing (scRNA-seq) data predicted by PRTS. (A) Uniform manifold approximation and projection (UMAP) visualization of cell clusters generated by PRTS based on the H&E image. Cell types include the following: Neu, neurons; OLG, oligodendrocytes; AC, astrocytes; CPEC, choroid plexus epithelial cells; GAC, a subpopulation similar to glutamatergic astrocytes; and VLM, vascular and leptomeningeal cells. (B) Dot plot showing the expression of marker genes across cell subtypes generated by PRTS. (C) Spatial distribution of manually annotated cell subtypes based on PRTS. (D) Spatial distribution of manually annotated cell subtypes based on ground truth sequencing data. (E) Localization of selected regions used for detailed visualization in (F) to (H). (F to H) Local distribution of PRTS-predicted cell subtypes in specific regions. Red lines delineate boundaries between distinct cell zones. (F) Cortex region. (G) Lateral ventricle region. (H) Hippocampus region. (I) Proportion of each cell subtype. Top, predicted data; bottom, ground truth data. (J) Proportion of major cell types. Left, predicted data; right, ground truth data. (K) Confusion matrix based on cell categories.

Since the smallest spatial unit for gene expression data generation is a single cell, the model enables us to directly extract single-cell subtypes from the predicted results. In this case, we have extracted 21 subtypes of cells in total, with a silhouette coefficient of 0.0952 and an adjusted rand index of 0.1424 (Fig. 3A and Table S5). They were annotated as one of the following types: neurons, astrocytes, oligodendrocytes, choroid plexus epithelial cells, vascular and meningeal cells, or glutamatergic astrocytes (Fig. 3A to C). Predicted results show agreement with the manual annotation (Fig. 3D). Cell annotation was performed by comparing the highly expressed genes of each virtual cell subtype with curated single-cell marker databases, including PanglaoDB [20] and CellMarker [21] (Table S5).

In the predicted results, different cell subtypes illustrated distinctive gene expression patterns (Fig. 3B). For neurons, we scored the marker gene sets for each subtype and found that the scores for different subtypes exhibited spatially specific distributions, clearly identifying anatomical structures such as the hippocampus and striatum. This suggests that the clustering results of neurons might be based on spatially differential gene expression across the anatomic structures (Fig. S7). The predictions for other cell subtypes even showed more typical expression features compared to neurons. Three oligodendrocyte subtypes universally exhibited high expression of the myelin-associated proteins *Mog* and *Mobp*, as well as the key transcription factors *Sox10* and *Myrf* [22]. Astrocytes were characterized by the elevated expression of *Sparc* [23] and *Vim* [24]. Choroid plexus epithelial cells are marked by high expression of *Ttr* [25]. Vascular and meningeal cells were annotated by the high expression of *Ptgds* [16]. Interestingly, a specific cell subpopulation exhibited gene expression patterns characteristic of both neurons and astrocytes (Fig. 3B and Fig. S8). This profile is consistent with recently reported glutamatergic astrocytes [26] but could also potentially represent tissue tiles where astrocytes are co-located with neurons.

Cell subtypes have specific spatial distributions that can be identified in both predicted results and ground truth measured by Visium HD (Fig. 3C to H). In the cortical region, different cell subtypes populated in specific regions and formed horizontal layers (Fig. 3F). In the external capsule and lateral ventricle regions, we captured an enriched population of oligodendrocytes and the presence of choroid plexus epithelial cells (Fig. 3G). In the hippocampal region, we identified the gradient distribution of various neuron subtypes (Fig. 3H), with Neu_01 predominantly enriched in the CA1 region and Neu_02 primarily enriched from the CA2 region to the dentate gyrus. The regional specification of cell subtypes has been verified by multiple ST atlases of the mouse brain [27,28]. This agreement suggests that PRTS is able to annotate brain tissue structures at subtype-level resolution. Quantitative comparisons further demonstrated near-identical proportions of neurons and rare subtypes relative to manual annotations, validating the framework’s accuracy in cell-type quantification (Fig. 3I and J). Due to differing cell clustering strategies, direct one-to-one correspondence between subpopulations was hindered. Therefore, we aggregated the subpopulations into broader categories and generated a confusion matrix. The results indicated an overall classification accuracy of 53.9% at the broad category level (Fig. 3K). We observed that the model exhibited suboptimal performance in identifying astrocytes, which may be attributed to their highly intricate morphological structure [29].

### PRTS improves the resolution of a spot-level ST method

**Fig. 4.**
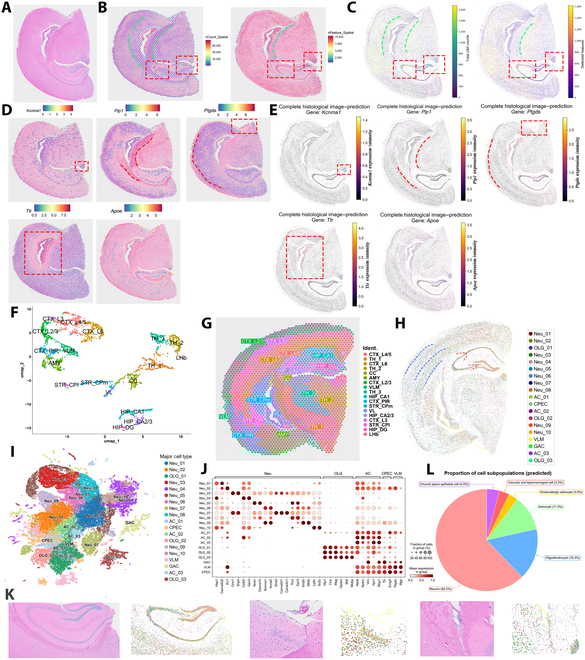
Analysis of spot-level spatial transcriptomics data predicted by PRTS. (A) Histological image corresponding to the spatial transcriptomics dataset (10x Genomics demo data). (B) Ground truth distribution of total counts (left) and number of detected features (right) across spatial transcriptomics spots. Each bright dot represents a spot. (C) PRTS-predicted total counts (left) and number of features (right) across the tissue section. For panels (B) and (C), red boxes highlight regions with high values, and a green line separates areas with contrasting value distributions. (D) Ground truth expression of selected genes across spatial transcriptomics spots. From left to right: *Kcnma1*, *Plp1*, *Ptgds*, *Ttr*, and *Apoe*. (E) PRTS-predicted gene expression at the single-cell level, for the same set of genes shown in panel (D). Each dot represents a cell. For panels (D) and (E), red boxes highlight high-expression regions, and a red line separates zones with contrasting expression patterns. (F) UMAP visualization of spot clusters. CTX, cortex; TH, thalamus; CC, corpus callosum; AMY, amygdala; VLM, vascular and leptomeningeal cells; HIP, hippocampus; PIR, piriform cortex; STR, striatum; CPm, medial caudate putamen; CPI, lateral caudate putamen; VL, lateral ventricle; DG, dentate gyrus; LHb, lateral habenula; L2/3, cortical layer 2/3; L3, layer 3; L4/5, layers 4/5; L6, layer 6. (G) Spatial distribution of spot clusters across the tissue section. (H) PRTS-predicted spatial distribution of cell subtypes across the section. Blue and red lines delineate distinct cellular zones within the cortex and hippocampus, respectively. (I) PRTS-predicted UMAP visualization of cell clusters. Neu, neurons; OLG, oligodendrocytes; AC, astrocytes; CPEC, choroid plexus epithelial cells; VLM, vascular and leptomeningeal cells; GAC, glutamatergic astrocytes. (J) Marker gene expression across predicted cell subtypes. (K) Regional views illustrating PRTS-predicted cell-type distributions. Each pair of panels shows a region of interest, with the left panel displaying the ground truth H&E staining image and the right panel showing the corresponding PRTS-predicted cell-type assignments. Regions from left to right: hippocampus, amygdala and adjacent meninges, lateral ventricle and corpus callosum. (L) Proportions of major predicted cell types.

Traditional spot-based ST technologies suffer from limited resolution and fail to resolve cellular heterogeneity. PRTS overcomes this limitation by generating transcriptomic profiles at single-cell resolution directly from histological images. In 2 spot-based ST datasets (2,298/2,234 spots), PRTS reconstructed transcriptomes for 62,711 and 60,259 cells, respectively, representing an approximately 27-fold increase in spatial resolution (Fig. 4 and Fig. S9).

It should be emphasized that, due to the limitations of current cell segmentation algorithms, the reported number may exceed the true number of cells as a result of artifacts and mis-segmentation. Therefore, quality control of the generated data is essential, such as removing certain cell subpopulations with only a small number of aggregated cells, which are likely to be mis-segmented tiles.

Building on PRTS’s ability to improve predictive accuracy, we further validated its performance on spot-based ST datasets. Overall, the predicted total cell counts and features aligned with actual spot distribution (Fig. 4B and C). For the prediction of individual genes, we visualized the same set of genes as in Fig. 2. The areas of high expression in the predicted images matched those in the ground truth (Fig. 4D and E).

Next, we assessed PRTS’s capability for single-cell annotation of brain tissues. All extracted cells were clustered into 19 subtypes, annotated as one of the following: neurons, oligodendrocytes, astrocytes, choroid plexus epithelial cells, vascular and meningeal cells, or glutamatergic astrocytes (Fig. 4H to L). Spot-based ST, on its own, cannot achieve single-cell annotation without cell composition inference algorithms.

It is well known that in spot-based ST methods, tissue structures can be divided into different tissue domains based on the gene expression pattern difference between spots [30]. We found that the predicted results can still reflect the distribution characteristics of tissue domains by identifying the enriched regions of different cells. The hippocampus is divided into 3 domains based on real sequencing data, namely, CA1, CA2 to CA3, and DG (Fig. 4G). In the predictions, the hippocampus was primarily occupied by Neu_08 (orange cluster), Neu_03 (green cluster), and Neu_02 (brown cluster), respectively (Fig. 4H). Similarly, both the measured and predicted data captured structural changes in multiple layers between the cortex and corpus callosum (Fig. 4H), highlighting the accuracy of PRTS. What sets PRTS apart is its finer, single-cell-level resolution, which allows us to zoom into local structures to identify cell distribution within tissue domains (Fig. 4K), offering deeper insights into the cellular microenvironment of specific regions.

### PRTS maintains robustness in cancer scenarios

Although we demonstrated the feasibility of PRTS in generating data from mouse brain samples, it remains necessary to evaluate its differences from existing algorithms and its potential applicability in broader contexts. One of the most important applications of ST and H&E imaging is cancer research and diagnosis, and many ST data generation algorithms have been developed specifically for cancer. To this end, we applied the PRTS framework to human breast cancer and lung cancer Visium HD datasets to assess its robustness in complex pathological tissues.

**Fig. 5.**
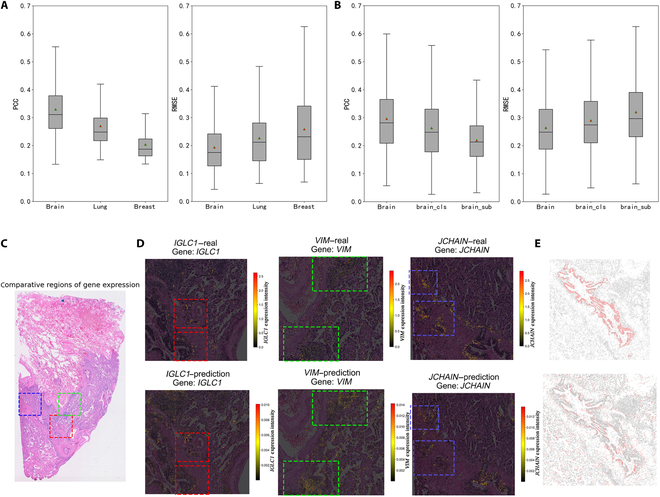
Performance of PRTS across different tissues. (A) Performance of PRTS on fresh-frozen mouse brain sections, human lung adenocarcinoma, and human breast cancer datasets. (B) Results of ablation experiments for PRTS on fresh-frozen mouse brain, human lung adenocarcinoma, and human breast cancer datasets. (C) Local regions selected for gene prediction in human lung adenocarcinoma. Red box, *IGLC1*; green, *VIM*; blue, *JCHAIN*. (D) Local prediction performance of PRTS in human lung adenocarcinoma. From left to right: *IGLC1*, *VIM*, and *JCHAIN*. For each gene, expression from the ground truth data (top panel) and the model prediction (bottom panel) are shown. Input data were normalized for model prediction. (E) Localized distribution of predicted cell subpopulations in human lung adenocarcinoma. Top: ground truth distribution, defined by one cell subgroup. Bottom: distribution predicted by PRTS, defined by 2 subgroups. PCC, Pearson correlation coefficient; RMSE, root mean square error.

First, we aimed to evaluate the regression accuracy of gene expression prediction across multiple tissue types. For breast cancer sections, a single slice was partitioned into different regions for independent model training and validation. For lung cancer sections, 2 consecutive slices were used separately for training and validation. In addition, we obtained a third fresh-frozen mouse brain section to further test predictive performance in the brain. We calculated the Pearson correlation coefficient (PCC) and root mean square error (RMSE) between predicted and true values—metrics commonly employed for evaluating predictive performance in similar algorithms. The results showed that across the mouse brain, lung cancer, and breast cancer, the mean PCC values for PRTS were 0.330, 0.270, and 0.204, respectively, while the mean RMSE values were 0.194, 0.227, and 0.259 (Fig. 5A). A complete list of PCC and RMSE values for each predictable gene is provided in Table S6 to facilitate detailed inspection of gene-level prediction performance.

It should be noted that although our algorithm, like many others, focuses on gene prediction, to the best of our knowledge, all mainstream ST prediction methods have been developed for conventional spot-level data rather than the single-cell-level ST used in our study. In other words, those algorithms generate RNA counts for entire spot regions, which include nuclear RNA, cytoplasmic RNA, extracellular RNA, and others, whereas our method quantifies only nuclear RNA. Previous studies have reported that nuclear RNA abundance does not directly correspond to total cellular RNA. For example, in the MIN6 pancreatic β-cell line, most genes were found to have more transcripts in the cytoplasm than in the nucleus [31]. Therefore, our algorithm cannot be directly compared on the same scale with other methods. To better contextualize performance in the gene regression task, we reviewed the predictive values reported in the literature. For average PCC, most reported values across samples fall between 0.1 and 0.4 [8,9,32–34]. Given that single-cell ST data are inherently more sparse and present greater challenges for numerical prediction, we consider the performance of PRTS in this task to be comparable to, and acceptable within, the range of existing methods.

Next, we conducted ablation experiments to evaluate the contributions of the local and global modules in complex tissue prediction. Specifically, we used the complete model as the baseline and systematically removed the features extracted from local pixel patches and global pixel patches, ensuring that the observed performance changes were attributable to the removed component. The results showed that removing either component led to a decline in predictive performance (Fig. 5B), demonstrating the overall effectiveness of our architectural design.

Finally, using lung cancer as an example, we further illustrated the mapping of normalized data within tissue sections (Fig. 5C to E). We presented the predicted expression patterns of the *IGLC1*, *VIM*, and *JCHAIN* genes in selected regions (Fig. 5D), all of which exhibited expression characteristics consistent with tissue structure. We observed the localization of cell subpopulations clustered around the bronchus region. Similar spatial clustering patterns were noted for both the ground truth and predicted data (Fig. 5E). However, due to the complexity of cell subtypes within the tumor microenvironment, detailed functional annotation of these subpopulations remains challenging. It should be emphasized that, unlike normal tissues with well-defined spatial organization, cancer tissue slices exhibit a more disordered cellular arrangement. Therefore, incorporating additional cancer tissue slices for training will likely be necessary in future work to uncover novel spatial structures and gene expression features in such complex pathological contexts.

## Discussion

Subcellular-resolution ST data enable a more accurate and comprehensive interpretation of spatial heterogeneity at the cellular level. Hence, we developed PRTS, a novel method that utilizes easily accessible H&E images to effectively predict large-scale ST data, addressing the scarcity of high-resolution ST data. Furthermore, by comparing prediction results from different samples, we can facilitate the identification and screening of potential spatial discrepancies. This approach may diminish the necessity for expensive sequencing experiments, thereby decreasing experimental expenditures and advancing spatially contextualized biomedical research.

Most existing methods rely on spot-level ST data, whereas PRTS is designed for single-cell-level modeling, creating a fundamental distinction from established prediction algorithms such as iStar, HiST2ST, and IGI-DL. PRTS predicts nuclear RNA, while these other methods capture total RNA signals within a region, making their outputs difficult to align both numerically and biologically. Moreover, single-cell-level data differ markedly from conventional ST in terms of dimensionality, sparsity, and value range, further complicating direct comparisons. We attempted comparisons at the super-pixel level, but inconsistencies in measurement units and data aggregation strategies prevented meaningful alignment of data points across algorithms. Nevertheless, we reported the PCCs of PRTS alongside published results to provide researchers with a clearer understanding of the relative strengths and limitations of the method.

By benchmarking the accuracy of predicted gene expression level and cell annotation with ground truth dataset, we showed that PRTS is capable of generating accurate subcellular ST data with real histological images. The performance of PRTS for brain slices is consistent with those in existing studies [27,28,35,36]. The algorithm can only predict ST data based on HVGs. The number of such genes is substantially lower than the total number of genes measurable by high-throughput sequencing platforms, which may result in incomplete or missing coverage of certain biologically relevant pathways. This limitation arises because most genes exhibit weak spatial associations that may not be reflected in image structures, and including an excessive number of genes would introduce tremendous computational burdens and exacerbate high-dimensional sparsity. In future work, we plan to employ approaches such as module detection to shift from predicting the expression of individual genes to inferring the activity of gene modules or biological pathways with coordinated functions, thereby enhancing the biological interpretability of the predictions.

We also recognize that further experimental validation, such as quantitative comparisons with IHC results, is still lacking. True IHC images may display diffuse positivity, making direct comparisons with predicted images challenging. Moreover, positivity criteria vary across methods, making quantitative analysis of such comparisons difficult. In particular, gene expression values at the single-cell level are distributed continuously, and defining positivity based on arbitrary thresholds can introduce observer subjectivity.

The predictive workflow in this study is built upon accurate single-cell segmentation. However, segmentation algorithms inherently produce errors, which may contribute to prediction inaccuracies—for example, splitting a single cell into multiple tiles or misclassifying artifacts as cells. As a result, the number of segmented cell tiles may exceed the true number of cells. To mitigate the impact of segmentation errors, we performed quality control by excluding abnormal tiles such as multicell aggregates. In the future, we plan to compare different segmentation algorithms to identify the most suitable approach for various tissue types [37]. Importantly, the PRTS framework itself is not dependent on any specific segmentation method; its core predictive module requires only cell images and coordinate information as input. Thus, the framework is inherently flexible and compatible with future advances in segmentation algorithms.

Moreover, early ST technologies had poor resolution for mixed expression information from multiple cells within each sequenced spot [38]. Therefore, methodologies such as deconvolution are necessary to annotate cell subtypes within individual spots [39,40]. Given the variability in performance among different deconvolution methods, the precision of cell-type annotation remains moderate [41].

Our current results demonstrate that PRTS can directly generate data at single-cell resolution and numerically impute values for regions that are difficult to sequence, thereby contributing to practical research applications. Nevertheless, we also observed that the average silhouette coefficient of the virtual subpopulations was relatively low. Although this is consistent with the inherent characteristics of single-cell clustering—such as high-dimensional sparsity and the continuous or transitional nature of cell states—where silhouette coefficients are often low even for real single-cell subpopulations [42,43], we remain cautious and plan to further optimize the algorithm to improve model performance. Furthermore, we found that the model’s ability to identify astrocytes was compromised. This limitation may be attributed to the complex morphology of astrocytes and their frequent close proximity to neurons [44]. Therefore, further validation with large-scale training datasets is required to improve the identification of specific and rare cell types.

In terms of data expansion, beyond mouse brain tissue, this study also incorporated human breast cancer and lung cancer Visium HD datasets. The results showed that PRTS maintained robust performance across different tissue types, providing preliminary evidence of its ability to capture the intrinsic anatomical features of diverse tissues. However, models trained from single sections still require validation in larger cohorts to confirm their generalizability. While PRTS was able to identify the gradient distributions and nuclear gene expression patterns of cells, this currently serves only as proof of concept. Drawing conclusions about novel spatial structures based on such limited data would lack robustness. Future studies will therefore focus on integrating multisample, large-scale ST datasets to build comprehensive models capable of systematically uncovering spatial functional units and heterogeneous patterns that are blurred in low-resolution ST data.

In addition, unavoidable technical deformations arise during tissue section preparation. The mouse brain sections used in this study were obtained through different preparation methods, including the formalin-fixed paraffin-embedded (FFPE) process, frozen fixation, and fresh-frozen processing. The morphological differences among these sections are far greater than those between simple consecutive sections, providing preliminary evidence that the algorithm exhibits a degree of tolerance to tissue deformation. Nevertheless, further evaluation is needed to assess the performance of PRTS across a wider range of section preparation conditions.

The biggest limitation is that, despite being a state-of-the-art ST technology, Visium HD has a limited number of slices available for training. Based on the current data scale, PRTS is not yet sufficient to support highly variable cross-species or cross-platform analyses. Different slice preparation techniques may lead to substantial morphological variation, impairing the high-accuracy prediction capability of PRTS. Expanding the types and scope of Visium HD sequencing, optimizing cell segmentation algorithms, and increasing the scale of training datasets are all crucial for developing a generic prediction model. More importantly, we have provided a framework for the development of single-cell-level ST data generation models.

Our team is actively collecting large cohorts of ST sections for single diseases such as lung cancer, through both mining of public datasets and prospective sequencing efforts. These cohorts encompass data from multiple platforms, including Xenium and Stereo-seq. The goal is to leverage the diversity of these sections to overcome the limitations of cross-sample heterogeneity, thereby constructing a clinically oriented, generalizable PRTS framework and further exploring its potential applications in tumor spatial biology.

## Methods

### Transcriptomics data preprocessing

All Visium HD datasets used in this study come from publicly available datasets provided by 10x Genomics (https://www.10xgenomics.com/datasets?configure%5BhitsPerPage%5D=50&configure%5BmaxValuesPerFacet%5D=1000&query=HD). The FFPE slides of the coronal region of the C57BL/6 mouse brain were downloaded as our training dataset, while the validation dataset consists of frozen-fixed slides of the coronal region of the C57BL/6 mouse brain. We extracted the 2 × 2-μm filtered barcode matrix, parquet tissue position matrix, and high-resolution histology feature images of the slices from all datasets.

To create nucleus masks for each cell in the slices, we began by performing cell segmentation on the high-resolution H&E images corresponding to the sequencing data. Images were subjected to percentile normalization before generating the nuclei segmentation masks. Cell segmentation was performed with StarDist2 [45], which identifies cell boundaries and generates polygon masks. We converted the results into GeoDataFrame using the GeoPandas package (v0.12) to record the spatial coordinates of each cell [46]. After the segmentation, we counted the sum of the unique barcodes within the nuclei regions. Cells meeting the following criteria were retained. For a single cell, (a) the value is less than 2,000, (b) the UMI count is greater than 20, and (c) the mitochondrial gene amount is below 15%. Finally, 56,761 cells in total were used for training. For the validation dataset, the same filtering criteria were applied, resulting in 77,602 cells.

Notably, when analyzing the new histological images, cell segmentation must be performed to obtain nucleus masks necessary for generating gene expression data. Since the current model was developed based on hemisection images, the full-brain H&E image was manually split prior to prediction. Our cell segmentation strategy and workflow were based on the analysis guidelines provided by 10x Genomics. See details at https://www.10xgenomics.com/analysis-guides/segmentation-visium-hd.

To understand the overall features of the cells in the training dataset, we performed dimensionality reduction and clustering on the dataset using Scanpy (v1.9.8) [47]. We identified 2,000 HVGs using the highly_variable_genes function. The top 50 principal components (PCs) were calculated using the pca function. Based on these principal component analysis (PCA) components, we constructed a neighborhood graph with the neighbors function and performed Leiden clustering on this graph (resolution = 0.6). Uniform manifold approximation and projection (UMAP) was used for dimensionality reduction.

Through the clustering process, we identified a total of 20 cell clusters. Differential gene expression analysis was carried out using the rank_genes_groups function in Scanpy. The results were tested with the Wilcoxon rank-sum test. We retained only genes with *P*.adjust < 0.01 and |log_2_FC| ≥ 1 for training, which resulted in a total of 1,820 genes. The motivation of identifying differentially expressed genes is to establish the correlation between the gene expression profiles and spatially morphological features of cells, which provides potential biomarkers for studying functional heterogeneity in cells.

Based on typical marker genes, we classified the cell clusters into 4 main types: neurons (*Snap25*, *Camk2n1*, *Ncdn*, and *Atp1b1*), oligodendrocytes (*Plp1*, *Mbp*, and *Mobp*), astrocytes (*Apoe* and *Aldoc*), and choroid plexus epithelial cells (*Clu*, *Ttr*, and *Enpp2*). Meanwhile, we also noticed a few cell clusters lacking distinct gene expression patterns. Given that subtypes without distinct expression profiles are commonly observed in both ST and single-cell RNA sequencing studies [48], these subtypes may represent a cluster of misidentified or low-quality cell spots. Thus, these clusters were included during the training process. When analyzing new slices, cells lacking distinct expression features tend not to be classified into any major cell types, instead forming separate clusters, making them easy to exclude.

In addition to the mouse brain datasets, lung and breast cancer tissue sections were processed using the same pipeline to assess generalizability. Following differential gene expression analysis and filtering, 1,656 genes were retained for the lung cancer model and 1,147 for the breast cancer model.

### Cellular gene expression prediction with histological images

To facilitate the processing of histological images with different resolutions, we first rescaled each image so that the size of each pixel is 0.5 × 0.5 μm^2^. This rescaling ratio ensures that a 16 × 16-pixel tile corresponds to an area of 8 × 8 μm^2^, which is about the size of a single cell [8]. Then, based on the cell masks obtained from cell segmentation, we drew a minimum bounding rectangle to the adjacent polygons to extract individual cell regions and extracted a 256 × 256-pixel tile from the histological image to serve as the cell’s neighboring spatial region. Additionally, for each cell, we divided the 256 × 256-pixel tile into 16 × 16-pixel patches.

The 16 × 16-pixel images primarily capture the fine-grained cellular structures, such as cell morphology and subcellular components, whereas the 256 × 256-pixel images reflect the global tissue structure. Let $X\in {\mathbb{R}}^{M\times N\times 3}$ be the RGB-channel histological image with a height M and a width N. For each cell, a 256 × 256-pixel image tile centered on its spatial location was extracted and used as input to a pre-trained vision transformer (ViT) model [49]. Specifically, we employed a ViT-256/16 model pre-trained using the self-supervised DINO framework [50], processing the image by dividing it into a sequence of 16 × 16 patches (tokens). The final hidden state of the dedicated [CLS] token serves as a holistic representation of the entire image tile, which we used as the global feature vector $C_{2}$.

To represent fine-grained cellular morphology, the local feature $C_{1}$ was derived from the patch tokens whose 16 × 16 regions overlapped with the cell nucleus. As a single cell typically spans multiple patches, their feature vectors were aggregated by average pooling to generate a unified $C_{1}$ vector. The local feature $C_{1}$ and the global feature $C_{2}$ were then concatenated to form a comprehensive histology feature vector $z_{x}\in {\mathbb{R}}^{C_{1}+C_{2}}$. In our implementation, the feature dimensions were set to $C_{1}=192$ and $C_{2}=384$.

In gene expression prediction tasks, the prevalence of zero values and sparse counts makes it challenging for conventional regression models to distinguish between low-value gene expression and zero expression. To address this, we designed a dual-output neural network model to simultaneously determine whether a gene is expressed and predicts its expression level. Given the class imbalance problem in transcriptomics datasets, where most genes are in a silent state and positive samples (active genes) are much fewer than negative samples (silent genes), we introduce class weights into the loss function to enhance the model’s capacity.

To express the loss function, let ${\hat{y}}_{i}$ be the gene expression prediction for gene i and $y_{i}$ be the observed gene expression for gene i. Then, the class weighting coefficient $\omega_{i}$ could be expressed numerically as

$$\omega_{i}=\left\{\begin{array}{cc} \omega_{p} & \text{if}\;y_{i}>0\\ \omega_{n} & \text{if}\;y_{i}=0 \end{array}\right.\;\text{where}\;\omega_{p}>\omega_{n}$$ (1)

Then, the weighted loss function is

$$L_{\mathrm{reg}}\left(\hat{y},\;y\right)=\sum_{i=1}^{d}w_{i}\cdot {\left({\hat{y}}_{i}-y_{i}\right)}^{2}$$ (2)

where d is the number of genes to predict, and the classification loss $L_{\mathrm{bin}}$ is calculated based on binary-classification labels of whether a gene is expressed or not. Then, the total loss function Loss is a weighted sum of these 2 losses:

$$\text{Loss}=\lambda_{1}L_{\mathrm{reg}}\left(\hat{y},\;y\right)+\lambda_{2}L_{\mathrm{bin}}\left(\hat{y},\;y\right)$$ (3)

The hyperparameters $\omega_{p}$, $\omega_{n}$, $\lambda_{1}$, and $\lambda_{2}$ were determined through grid search on the validation set to optimize performance. In this study, we set $\omega_{n}=1$, $\omega_{p}=5$, $\lambda_{1}=1$, and $\lambda_{2}=20$ based on the optimal validation results.

After model training, the predicted gene expression for gene i at cell x is

$${\hat{y}}_{x,i}=f_{\mathrm{MLP}}{\left(z_{x}\right)}_{i}$$ (4)

where $f_{\mathrm{MLP}}{\left(\cdot \right)}_{i}$ is the ith dimension of the output vector from the neural network model and $z_{x}\in R^{576}$ is the histology feature vector of cell x. The predicted expression values obtained for all cells were denormalized and integrated into a gene expression matrix, which was then exported as an .h5ad file for downstream analysis.

For the network architecture, we used a feed-forward neural network consisting of 4 hidden layers with 512, 512, 1,024, and 1,024 nodes, respectively. Each hidden layer employed a leaky rectified linear unit as the activation function, followed by a batch normalization layer to accelerate training and a dropout layer (rate = 0.1) to prevent overfitting. The output layer was a linear layer with the number of nodes equal to the number of predicted genes.

The model was trained using the AdamW optimizer with a learning rate of 0.01 and a weight decay of 0.05. Training was conducted on an NVIDIA GeForce RTX 4060 graphics processing unit with a batch size of 256 for 100 epochs, with early stopping applied if the validation loss did not improve for 10 consecutive epochs. The total number of model parameters was approximately 4.0 million. The entire analytical workflow, from histological image input to generation of the gene expression matrix, was completed within 100 min for a single dataset. Detailed runtimes for training and prediction are reported in Table S6.

### Evaluation criteria for gene expression prediction

To assess the accuracy of predicted gene expressions at the cellular level, we compared the predicted values with ground truth measurements for each gene across all cells. Both ground truth and predicted values were normalized using the log1p transformation. Prediction accuracy was evaluated using 2 primary metrics: RMSE and PCC.

RMSE was calculated by treating ground truth and predicted expression values as vectors and computing their euclidean distance. This metric provides a straightforward measure of the overall prediction error. However, because RMSE does not reflect the strength of linear associations, we also employed PCC to evaluate how well predicted expression levels captured the relative patterns of gene activity across cells.

PCC quantifies the linear correlation between 2 variables, offering insight into the model’s ability to rank expression levels across cells even when systematic differences in absolute value exist. In the context of cellular-resolution prediction, RMSE and PCC provide complementary perspectives: RMSE measures the magnitude of absolute error, while PCC assesses the consistency of relative patterns.

To examine the contribution of the multiscale features, we conducted an ablation study. Specifically, we compared the full model (incorporating both the local feature, $C_{1}$, and global features, $C_{2}$) against 2 reduced models: one using only local features ($C_{1}$) and another using only global features ($C_{2}$).

### Model visualization of gene expression patterns

We computed key quality metrics such as the total counts and the number of features for each cell spot using the pp.calculate_qc_metrics function in Scanpy. Image registration was applied to map the spatial location of cells with their coordinates as well as the quality metrics of the expression matrix. To visualize the spatial heterogeneity of total counts and features, we plotted a 3-panel figure using Matplotlib (v3.7.3), showing the original tissue slice, the heatmap of UMI counts, and the heatmap of the number of features [51]. We used the 99th percentile to set the color range to eliminate the influence of outliers. Similarly, we used the same aforementioned spatial mapping algorithm to label the individual gene expression intensity with the microanatomical structure. For multigene scoring, we utilized Scanpy’s built-in score_genes method to integrate and score target gene sets (the top 5 marker genes for each neuronal subtype) and labeled these scores with the microanatomical structure.

### Preparation of validated immunohistochemical sections

All relevant procedures involving animal experiments presented in this study were compliant with ethical regulations regarding animal research and were conducted under the approval of the Experimental Animal Ethics Committee of the South China University of Technology (license number AE-2025083).

Mouse brains were dissected from 6- to 10-week-old C57BL/6J male mice. The mice were housed at a relative humidity of 45% ± 15% and a room temperature of 23.0 ± 1.5 °C in the SPF Laboratory Animal Research Center of the South China University of Technology. Following the dissection, the brain tissues were fixed in 4% paraformaldehyde and incubated for 24 h at room temperature. The brain tissues were washed by phosphate-buffered saline (PBS) buffer to remove the excess paraformaldehyde and transported to Wuhan Servicebio Technology for paraffin embedding, H&E staining, and subsequent IHC.

To prepare FFPE samples, mouse brain tissue samples were fixed and trimmed, followed by graded ethanol dehydration, xylene clearing, and paraffin infiltration. The samples were then embedded using a paraffin embedding station. The FFPE blocks were sliced into 4-μm sections, flattened out in a 40 °C water bath, and dried at 60 °C.

Then, paraffin-embedded tissue sections were deparaffinized twice in xylene (20 min each), hydrated in pure ethanol (5 min), hydrated again in 75% ethanol (5 min), and gently washed in the water. The tissue sections on the slide were stained with hematoxylin and incubated for 3 to 5 min at room temperature. The slides were then treated with differentiation and bluing buffer. After dehydration in 85% and 95% ethanol (5 min each), the slides were stained with eosin for 5 min. Final dehydration and clearing were performed using pure ethanol 3 times (5 min each) and xylene twice (5 min each). Slides were mounted with neutral resin and imaged with a microscope.

For IHC, paraffin-embedded tissue sections were deparaffinized 3 times (10 min each), followed by rehydration for 3 times (5 min each), and then rinsed with distilled water. Antigen retrieval was performed under conditions specified in Table S4, followed by 3 washes with PBS (pH 7.4) for 5 min each. Endogenous peroxidase activity was blocked with 3% H_2_O_2_ for 25 min, followed by PBS wash and blocking with 3% bovine serum albumin for 30 min. Primary antibodies were applied and incubated overnight at 4 °C. After washing with PBS, slides were incubated with horseradish peroxidase-conjugated secondary antibodies at room temperature for 50 min. 3,3′-Diaminobenzidine was used for chromogenic development, followed by hematoxylin staining. Slides were dehydrated through a gradient alcohol (75% ethanol → 85% ethanol → pure ethanol for twice → *n*-butanol → xylene, 5 min each). The slides were mounted with neutral resin, examined, and imaged under a microscope.

### Cell annotation and spatial mapping

This study employed a systematic ST analysis workflow to annotate the predicted data. First, the raw data matrix generated by PRTS was normalized using the Scanpy framework (including total count normalization and log1p transformation). After filtering with the highly_variable_genes function, 800 HVGs were retained. The top 50 PCs were computed using the pca function. A neighborhood graph based on PCA components was generated using the neighbors function, followed by Leiden clustering with a resolution of 0.6. When annotating the ground truth dataset, the resolution for Leiden clustering was increased to 1.8 to ensure a comparable number of clusters. UMAP was employed for dimensionality reduction. In the annotation process, only cell clusters with a cell count of 1,200 or greater were retained. This step helped remove low-quality and misidentified image tiles. For the retained cell subtypes, differential expression analysis was performed using the Wilcoxon rank-sum test to identify subtype-specific marker genes. A threshold of |log_2_FC| ≥ 0.25 was used for screening. For the subsequent annotation step, only the top 25 most differentially expressed genes were included. Cell types were annotated based on the biological functions and spatial differential expression patterns of the marker genes.

We primarily referenced 2 databases: PanglaoDB and CellMarker. PanglaoDB evaluates the expression of specific genes across different cell types using 1,368 single-cell transcriptome datasets, whereas CellMarker provides manually curated markers for 9,148 cell types across various mouse tissues. For most virtual subpopulations, the highly expressed genes we identified could be supported by evidence from these databases.

For gene enrichment analysis, we selected genes with |log_2_(FoldChange)| > 0 and converted the selected gene list from gene symbols to UniProt. Gene Ontology enrichment analysis was performed using goatools (v1.4.12) with a *P* value set to 0.2 [52]. The Benjamini–Hochberg method was used to control the false-positive rate, and biological processes, molecular functions, and cellular components were analyzed separately.

In the validation slices, we performed further clustering and annotation on the cell clusters generated from the ground truth dataset. The methods and parameters used for clustering were kept the same as those for the predicted data, except for the resolution of the Leiden clustering.

In the quantitative analysis, we used Seaborn (v0.13.2) to plot horizontal bar charts that display the proportion of each cell subtype relative to the total cell count [51]. Pie charts were utilized to visualize the proportion of major cell types out of the total cell population.

To evaluate performance at the broad cell category level, the 21 annotated cell subpopulations were consolidated into 4 major classes when generating the confusion matrix: all neuronal subpopulations (including the putative GAC subpopulation due to its distinct glutamatergic expression profile) were grouped under “Neurons”, oligodendrocyte subpopulations were aggregated into “Oligodendrocytes”, astrocyte subpopulations were combined as “Astrocytes”, and cells such as choroid plexus epithelial cells (CPEC) and vascular and leptomeningeal cells (VLM) were merged into “Stromal Cells”.

Based on the characteristics of the lung cancer data, the following adjustments were made during annotation: for the original data, the resolution parameter for Leiden clustering was set to 0.5, and clusters containing fewer than 2,000 cells were removed; for the predicted data, the resolution was set to 0.6, and clusters with fewer than 1,200 cells were filtered out.

### Standard analysis of spot-level ST

The spot-level ST data used in this study come from 2 publicly available 10X Visium datasets provided by 10x Genomics. All datasets consist of coronal sections of mouse brains prepared by the FFPE process. We extracted the filtered expression matrices in HDF5 format and the corresponding spatially resolved images for standard ST analysis. Additionally, we extracted high-resolution histological images for generating prediction results.

The standard ST analysis pipeline was implemented in R (version 4.2.1) using Seurat (v5.1.0) to analyze the extracted data [53]. A spot was retained for further analysis if it met the criteria: a total gene count between 1,000 and 100,000 and a number of expressed genes between 200 and 10,000. After filtering, the 2 datasets retained 2,298 (Fig. 4) and 2,234 (Fig. S9) spots, respectively.

We normalized the ST data using the SCTransform method and performed PCA with the RunPCA function. The FindNeighbors function was employed to construct a neighborhood graph based on the PCA components. For the 2-dimensional visualization of spot clusters, the RunUMAP function was used to create a UMAP plot. The top 30 PCs were filtered for computing the embeddings. Ultimately, we identified 18 and 16 spot clusters in 2 datasets (Fig. 3 and Fig. S8), respectively.

### Analysis of spot-level ST using PRTS

We began with converting the high-resolution histological images corresponding to TIFF format and then followed the same method and parameters as outlined in the “Transcriptomics data preprocessing” section to perform cell segmentation. It is important to keep the H&E images used for actual prediction as similar as the images in the training dataset, so we must select images that are complete hemisphere slices and follow an identical orientation for slice placement, with the hippocampal area located at the top right corner of the overall tissue image.

Once the cell segmentation results were loaded into PRTS and the output cell X gene expression matrices were obtained, we evaluated the accuracy of the predicted gene expression values and cell annotations following the same steps as for slices in the validation datasets. In terms of gene expression, we used Scanpy to compute the total counts and number of features for each cell spot, labeling this information with the spatial coordinates of the cells. Similarly, we employed the same spatial mapping algorithm to label single-gene expression intensities with the microanatomical structure. For cell annotation, we first performed normalization on the raw data matrix and retained 600 HVGs using the highly_variable_genes function. The top 50 PCs were computed with the pca function. We used the neighbors function to plot neighborhood graphs based on these PCA components and performed Leiden clustering with a resolution of 0.6 on them. Similarly, only clusters with 1,200 or more cells were retained.

At this resolution, we identified 19 and 17 cell subtypes in 2 datasets, respectively (Fig. 4 and Fig. S9). Differential expression analysis was performed using the Wilcoxon rank-sum test, with |log_2_FC| ≥ 0.25 as the threshold. The top 25 most differentially expressed genes were used for cell annotation.

### Statistical analysis

The statistical analysis was implemented in R, RStudio, and Python. Differences were considered significant when *P* < 0.05.

The code of PRTS is available at https://github.com/morkwok/PRTS.

## Data Availability

We analyzed the following publicly available ST datasets: (a) Visium HD Spatial Gene Expression Library, Mouse Brain (FFPE) (https://www.10xgenomics.com/datasets/visium-hd-cytassist-gene-expression-libraries-of-mouse-brain-he); (b) Visium HD Spatial Gene Expression Library, Mouse Brain (Fixed Frozen) (https://www.10xgenomics.com/datasets/visium-hd-cytassist-gene-expression-mouse-brain-fixed-frozen); (c) Mouse Brain Coronal Section 1 (FFPE) (https://www.10xgenomics.com/datasets/mouse-brain-coronal-section-1-ffpe-2-standard); (d) Mouse Brain Coronal Section 2 (FFPE) (https://www.10xgenomics.com/datasets/mouse-brain-coronal-section-2-ffpe-2-standard); (e) Visium HD Spatial Gene Expression Libraries, Post-Xenium, Human Lung Cancer (FFPE) (https://www.10xgenomics.com/datasets/visium-hd-cytassist-gene-expression-human-lung-cancer-post-xenium-expt); (f) Visium HD Spatial Gene Expression Library, Mouse Brain (Fresh Frozen) (https://www.10xgenomics.com/datasets/visium-hd-cytassist-gene-expression-mouse-brain-fresh-frozen); and (g) Visium HD Spatial Gene Expression Library, Human Breast Cancer (Fixed Frozen) (https://www.10xgenomics.com/datasets/visium-hd-cytassist-gene-expression-human-breast-cancer-fixed-frozen).
