# Supplementary material for: PRTS: Predicting Single-Cell Spatial Transcriptomic Maps from Histological Images
Source: Research (Wash D C). 2025 Nov 6;8:0961. doi: 10.34133/research.0961 (PMC12589771; doi:10.34133/research.0961)
Supplement: Supplementary 1 — Figs. S1 to S9 Tables S1 to S6 [file research.0961.f1.zip › S1.pdf]

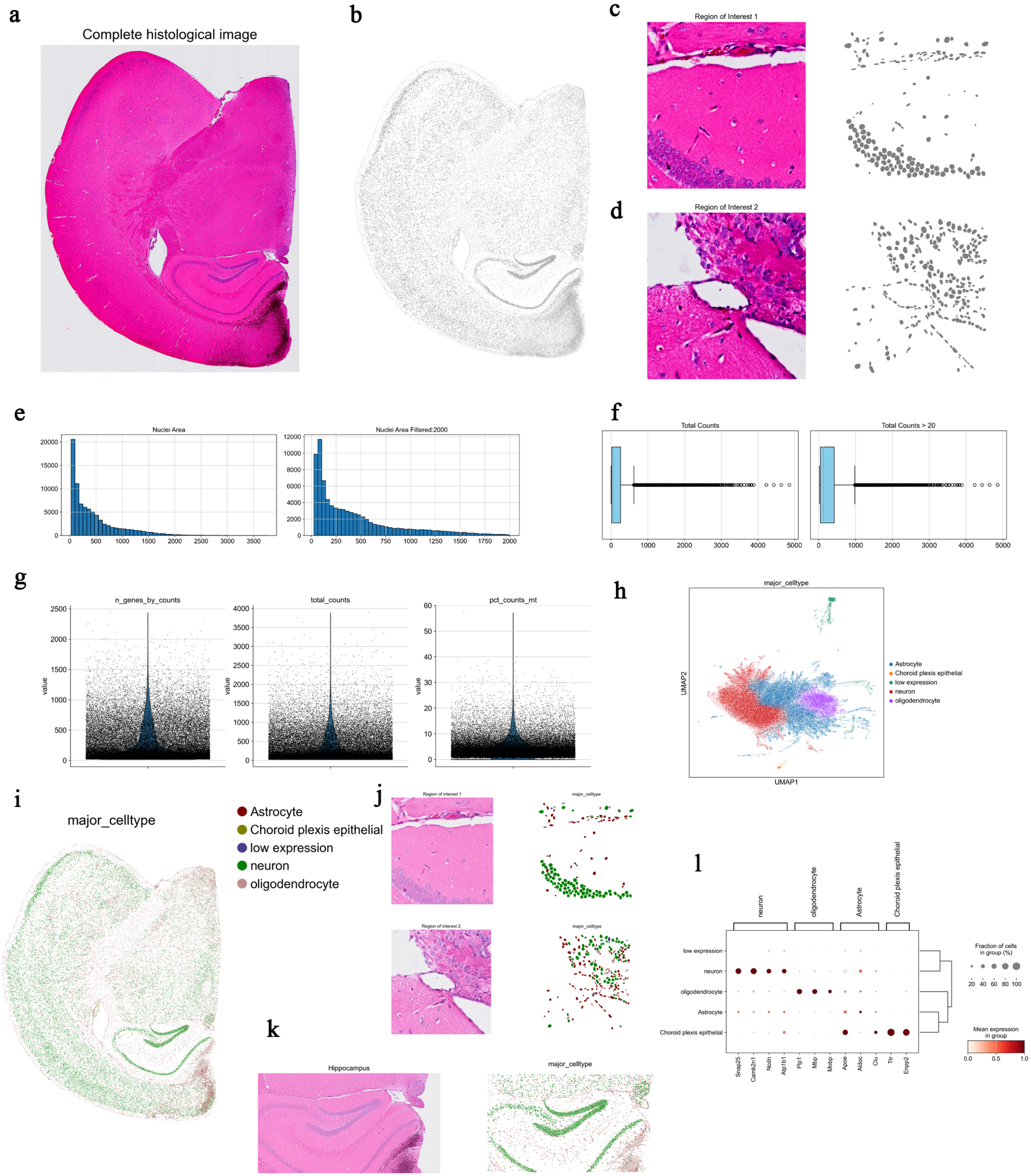

a
Complete histological image
b
c
Region of Interest 1
d
Region of Interest 2
e
Nuclei Area
Nuclei Area Filtered:2000
f
Total Counts
Total Counts > 20
g
n_genes_by_counts
total_counts
pct_counts_mt
value
h
major_celltype
UMAP1
UMAP2
Astrocyte
Choroid plexis epithelial
low expression
neuron
oligodendrocyte
i
major_celltype
Astrocyte
Choroid plexis epithelial
low expression
neuron
oligodendrocyte
j
Region of interest 1
major_celltype
Region of interest 2
major_celltype
k
Hippocampus
major_celltype
l
neuron
oligodendrocyte
Astrocyte
Choroid plexis epithelial
low expression
neuron
oligodendrocyte
Astrocyte
Choroid plexis epithelial
Snap25
Camk2n1
Ncdn
Atp1b1
Plp1
Mbp
Mobp
Apoe
Aldoc
Clu
Ttr
Enpp2
Fraction of cells in group (%)
20 40 60 80 100
Mean expression in group
0.0 0.5 1.0
