# Supplementary material for: PRTS: Predicting Single-Cell Spatial Transcriptomic Maps from Histological Images
Source: Research (Wash D C). 2025 Nov 6;8:0961. doi: 10.34133/research.0961 (PMC12589771; doi:10.34133/research.0961)
Supplement: Supplementary 1 — Figs. S1 to S9 Tables S1 to S6 [file research.0961.f1.zip › S3.pdf]

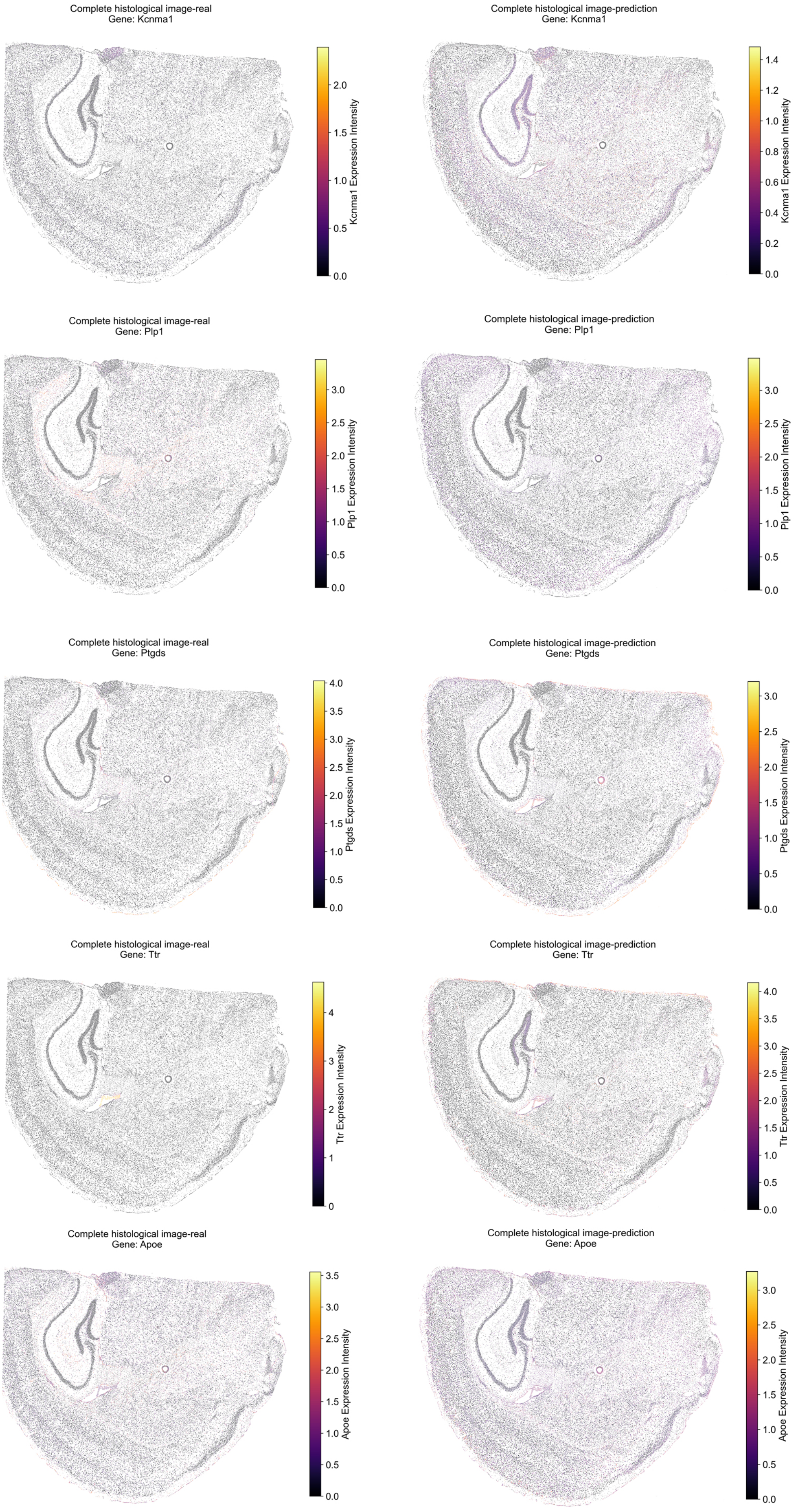
Complete histological image-real
Gene: Kcnma1
Kcnma1 Expression Intensity
0.0 0.5 1.0 1.5 2.0
Complete histological image-prediction
Gene: Kcnma1
Kcnma1 Expression Intensity
0.0 0.2 0.4 0.6 0.8 1.0 1.2 1.4
Complete histological image-real
Gene: Plp1
Plp1 Expression Intensity
0.0 0.5 1.0 1.5 2.0 2.5 3.0
Complete histological image-prediction
Gene: Plp1
Plp1 Expression Intensity
0.0 0.5 1.0 1.5 2.0 2.5 3.0
Complete histological image-real
Gene: Ptgds
Ptgds Expression Intensity
0.0 0.5 1.0 1.5 2.0 2.5 3.0 3.5 4.0
Complete histological image-prediction
Gene: Ptgds
Ptgds Expression Intensity
0.0 0.5 1.0 1.5 2.0 2.5 3.0
Complete histological image-real
Gene: Ttr
Ttr Expression Intensity
0 1 2 3 4
Complete histological image-prediction
Gene: Ttr
Ttr Expression Intensity
0.0 0.5 1.0 1.5 2.0 2.5 3.0 3.5 4.0
Complete histological image-real
Gene: Apoe
Apoe Expression Intensity
0.0 0.5 1.0 1.5 2.0 2.5 3.0 3.5
Complete histological image-prediction
Gene: Apoe
Apoe Expression Intensity
0.0 0.5 1.0 1.5 2.0 2.5 3.0
