# Supplementary material for: PRTS: Predicting Single-Cell Spatial Transcriptomic Maps from Histological Images
Source: Research (Wash D C). 2025 Nov 6;8:0961. doi: 10.34133/research.0961 (PMC12589771; doi:10.34133/research.0961)
Supplement: Supplementary 1 — Figs. S1 to S9 Tables S1 to S6 [file research.0961.f1.zip › Supporting Information.docx]

**Supplementary Fig. 1 | Data analysis of Visium HD training dataset**

(A) Full coronal tissue section from the Visium HD training dataset (10x Genomics demo data).

(B) Overview of cell segmentation; each dot represents the nuclear mask of a single cell.

(C-D) Local views of segmentation in selected tissue regions. Left panels show the raw H&E images; right panels display corresponding nuclear masks.

(E) Nuclear area distribution across cell tiles. Abnormally large areas may result from merged nuclei (e.g., cell doublets or multiplets). Only tiles with nuclear area <2000 were retained for downstream analysis. Left, before quality control (QC); right, after QC.

(F) Total UMI counts per cell tile. Cells with >20 UMIs were retained. Left, before QC; right, after QC.

(G) Distributions of the number of detected features, total counts, and mitochondrial gene content across cell tiles.

(H) Major cell types identified in the Visium HD dataset, visualized by UMAP.

(I) Spatial distribution of cell types across the tissue section.

(J-K) Local views of cell-type annotations. Panel j corresponds to the regions shown in c and d; panel k highlights the annotated hippocampal region. Panels j and k share the same color scheme as panel i.

(L) Dot plot showing expression of marker genes across major cell types.

**Supplementary Fig. 2 | Analysis of the Visium HD validation dataset.**

(A) Full coronal tissue section from the Visium HD validation dataset (10x Genomics demo data).

(B) Overview of cell segmentation; each dot represents the nuclear mask of a single cell.

(C-D) Local views of segmentation in selected regions. Left panels show the raw H&E images; right panels show the corresponding nuclear masks.

(E) Nuclear area distribution across cell tiles. Only tiles with nuclear area <2000 were retained for downstream analysis. Left, before quality control (QC); right, after QC.

(F) Total UMI counts per cell tile. Cells with >20 UMIs were retained. Left, before QC; right, after QC.

(G) Distributions of detected features, total counts, and mitochondrial gene content per cell tile.

**Supplementary Fig. 3 | Comparison of expression levels of specific genes in the tissue section.**

Left panel: Gene expression measured by Visium HD. Right panel: PRTS-predicted gene expression. From top to bottom: *Kcnma1*, *Plp1*, *Ptgds*, *Ttr*, *Apoe*. Each spot represents a cell.

**Supplementary Fig. 4 | Consecutive tissue sections used to evaluate model predictions.**

(A) H&E staining image.

(B) Predicted *Ptgds* expression based on panel a.
(C) Predicted *Apoe* expression based on panel a.

(D) H&E staining image.

(E) Predicted *Ptgds* expression based on panel d.

(F)Predicted *Apoe* expression based on panel d.

Panels a and d represent H&E images of the left and right hemispheres from the same mouse brain.

(G) IHC staining for Ptgds.
(H) IHC staining for Apoe.

**Supplementary Fig. 5 | Model predictions for 18 genes.**

For each gene, the left panel shows the ground truth expression, and the right panel shows the model prediction. Genes are displayed in the order listed in Supplementary Table 3. Each dot represents a single cell.

**Supplementary Fig. 6 | Model predictions for 18 genes**.

For each gene, the left panel shows the ground truth expression and the right panel shows the model prediction. Genes are displayed in the order listed in Supplementary Table 3. Each dot represents a single cell.

**Supplementary Fig. 7 | Spatial distribution of marker gene scores for 14 neuronal subtypes.**

For each subtype, the score was calculated using the top five marker genes. Subtypes are displayed in the order of their assigned suffixes. Each dot represents a single cell.

**Supplementary Fig. 8 | Ground truth-based cell annotation and GO enrichment comparison.**

(A) UMAP visualization of cell clusters manually annotated from sequencing data.
(B) Dot plot showing expression of marker genes across cell subtypes from sequencing data.

(C) GO enrichment results based on differentially expressed genes (DEGs) of glutamatergic astrocytes predicted by the model.
(D) GO enrichment results based on DEGs of glutamatergic astrocytes derived from ground truth data.

**Supplementary Fig. 9 | Analysis of spot-level spatial transcriptomics data predicted by PRTS, based on a dataset different from that used in Fig. 4.**

(A) Histological image corresponding to the spatial transcriptomics dataset (10x Genomics demo data).

(B) Ground truth distribution of total counts (left) and number of detected features (right) across spatial transcriptomics spots. Each bright dot represents a captured spot.

(C) PRTS-predicted total counts (left) and number of features (right) across the tissue section.

For panel b and c, red boxes highlight regions with high values, and a green line separates areas with contrasting value distributions.

(D) Ground truth expression of selected genes across spatial transcriptomics spots. From left to right: *Kcnma1*, *Plp1*, *Ptgds*, *Ttr*, and *Apoe*.

(E) PRTS-predicted gene expression at the single-cell level, for the same set of genes shown in panel d. Each dot represents a cell.

For panel d and e, red boxes highlight high-expression regions, and a red line separates zones with contrasting expression patterns.

(F) UMAP visualization of spot clusters. CTX, cortex; TH, thalamus; CC, corpus callosum; AMY, amygdala; VLM, vascular and leptomeningeal cells; HIP, hippocampus; PIR, piriform cortex; STR, striatum; CPm, medial caudate putamen; CPI, lateral caudate putamen; VL, lateral ventricle; DG, dentate gyrus; LHb, lateral habenula; L2/3, cortical layer 2/3; L3, layer 3; L4/5, layers 4/5; L6, layer 6.

(G) Spatial distribution of spot clusters across the tissue section.

(H) PRTS-predicted spatial distribution of cell subtypes across the section. Blue and red lines delineate distinct cellular zones within the cortex and hippocampus, respectively.

(I) PRTS-predicted UMAP visualization of cell clusters. Neu, neurons; OLG, oligodendrocytes; AC, astrocytes; CPEC, choroid plexus epithelial cells; VLM, vascular and leptomeningeal cells; GAC, glutamatergic astrocytes.

(J) Marker gene expression across predicted cell subtypes.

(K) Regional views illustrating PRTS-predicted cell-type distributions. Each pair of panels shows a region of interest, with the left panel displaying the ground truth H&E staining image and the right panel showing the corresponding PRTS-predicted cell-type assignments. Regions from left to right: hippocampus, amygdala and adjacent meninges, lateral ventricle and corpus callosum.

(L) Proportions of major predicted cell types.
