# Supplementary figures and images for: PRTS: Predicting Single-Cell Spatial Transcriptomic Maps from Histological Images
Source: Research (Wash D C). 2025 Nov 6;8:0961. doi: 10.34133/research.0961 (PMC12589771; doi:10.34133/research.0961)

**a**

Complete histological image

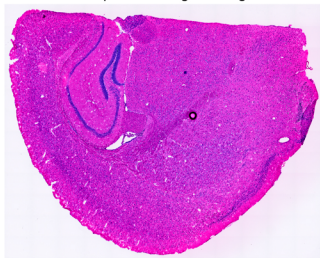**b**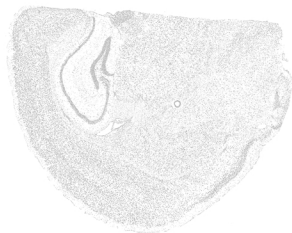**c**

Region of Interest 1

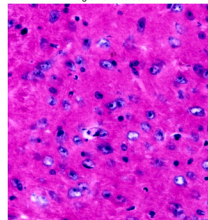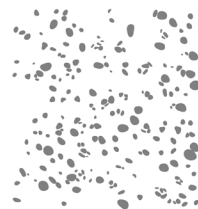**d**

Region of Interest 2

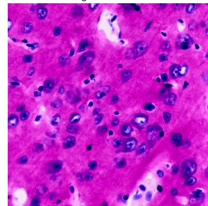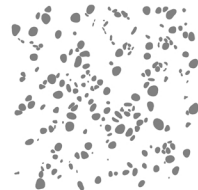**e**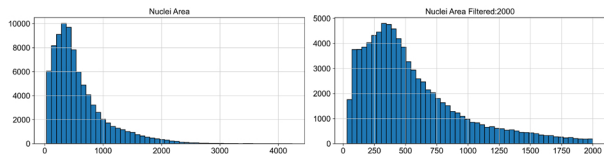**f**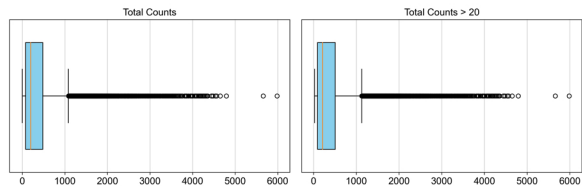**g**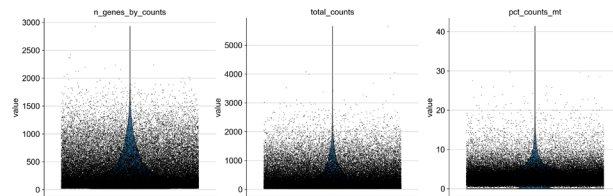

Supplement: Supplementary 1 — Figs. S1 to S9 Tables S1 to S6 [file research.0961.f1.zip › S2.pdf]

**a**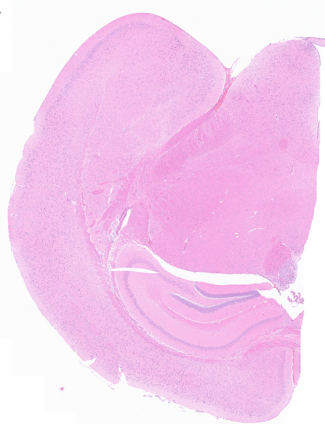**b**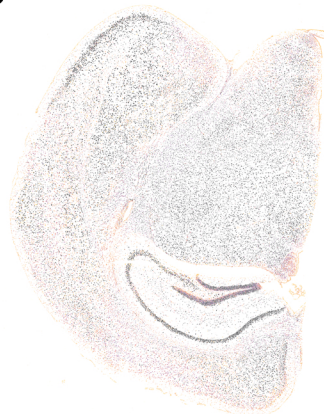**c**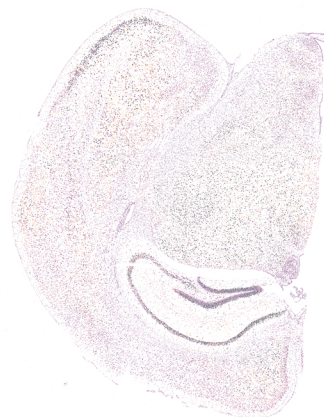**d**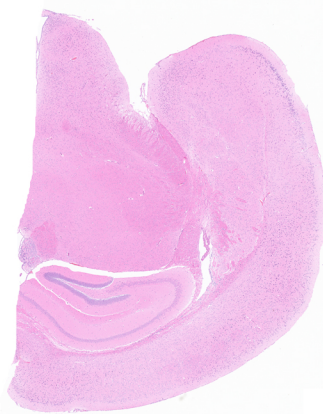**e**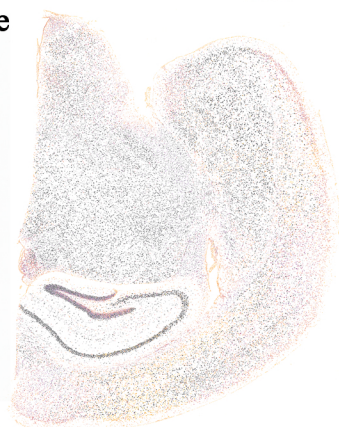**f**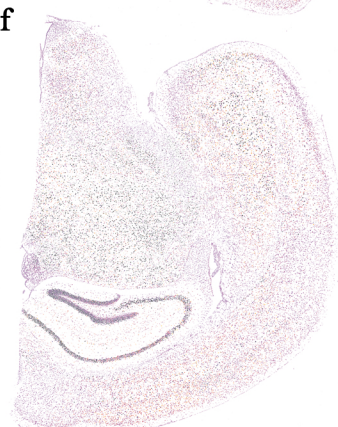**g**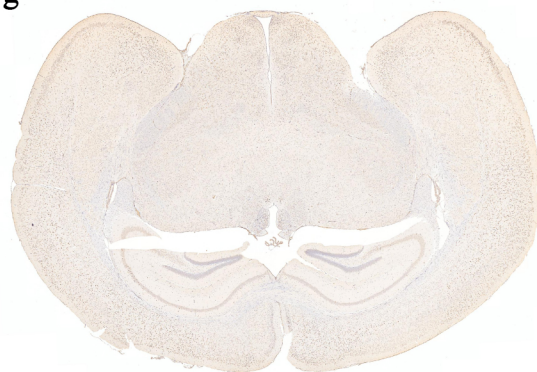

500  $\mu$ m

**h**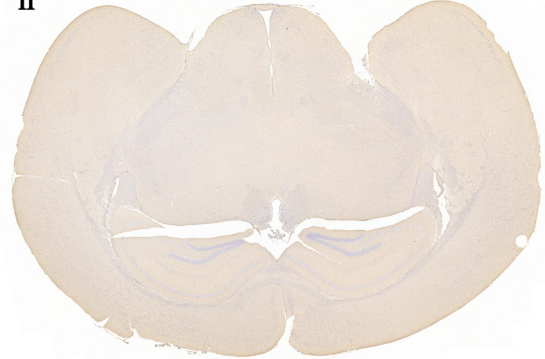

500  $\mu$ m

Supplement: Supplementary 1 — Figs. S1 to S9 Tables S1 to S6 [file research.0961.f1.zip › S4.pdf]

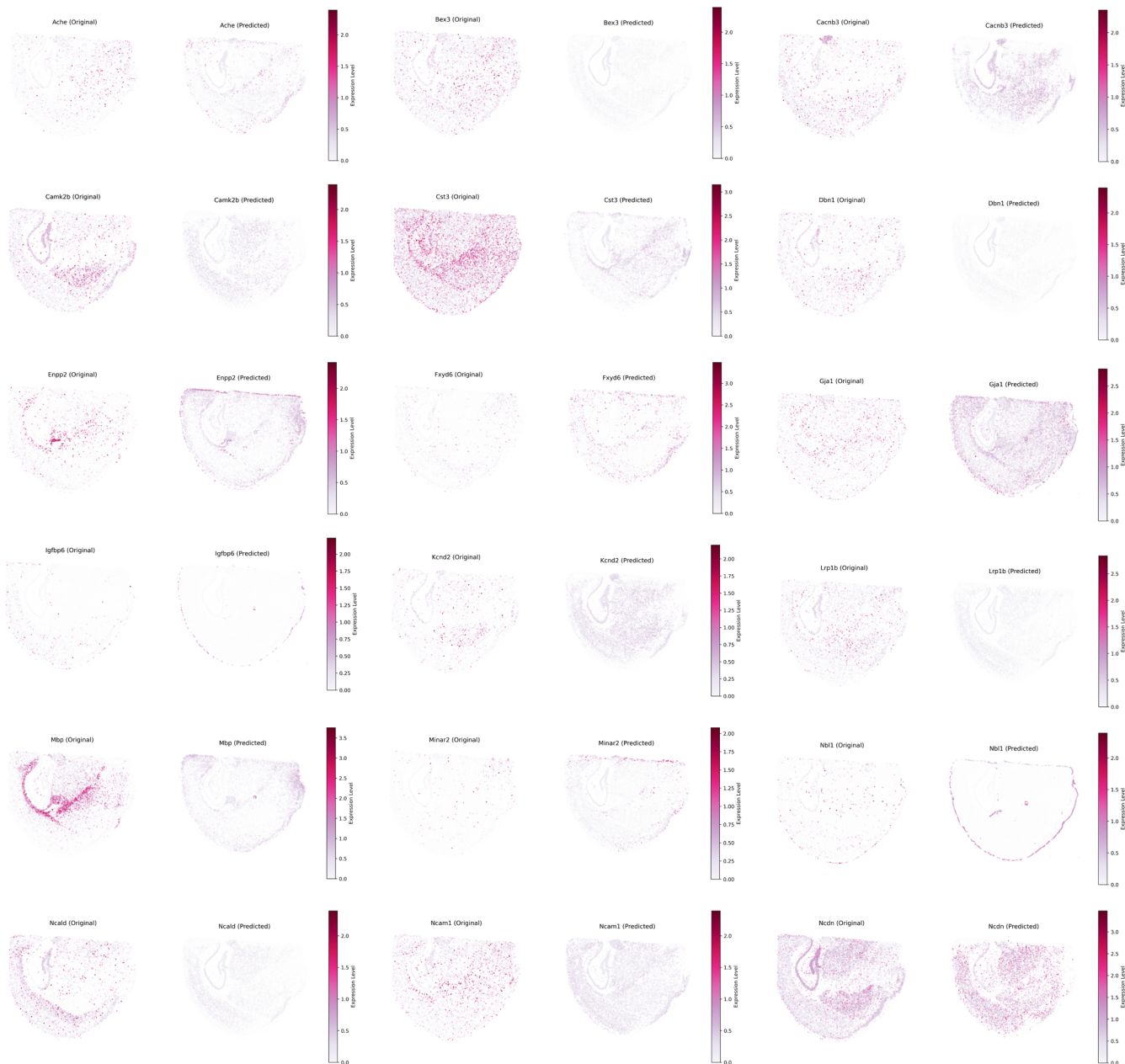

Supplement: Supplementary 1 — Figs. S1 to S9 Tables S1 to S6 [file research.0961.f1.zip › S5.pdf]

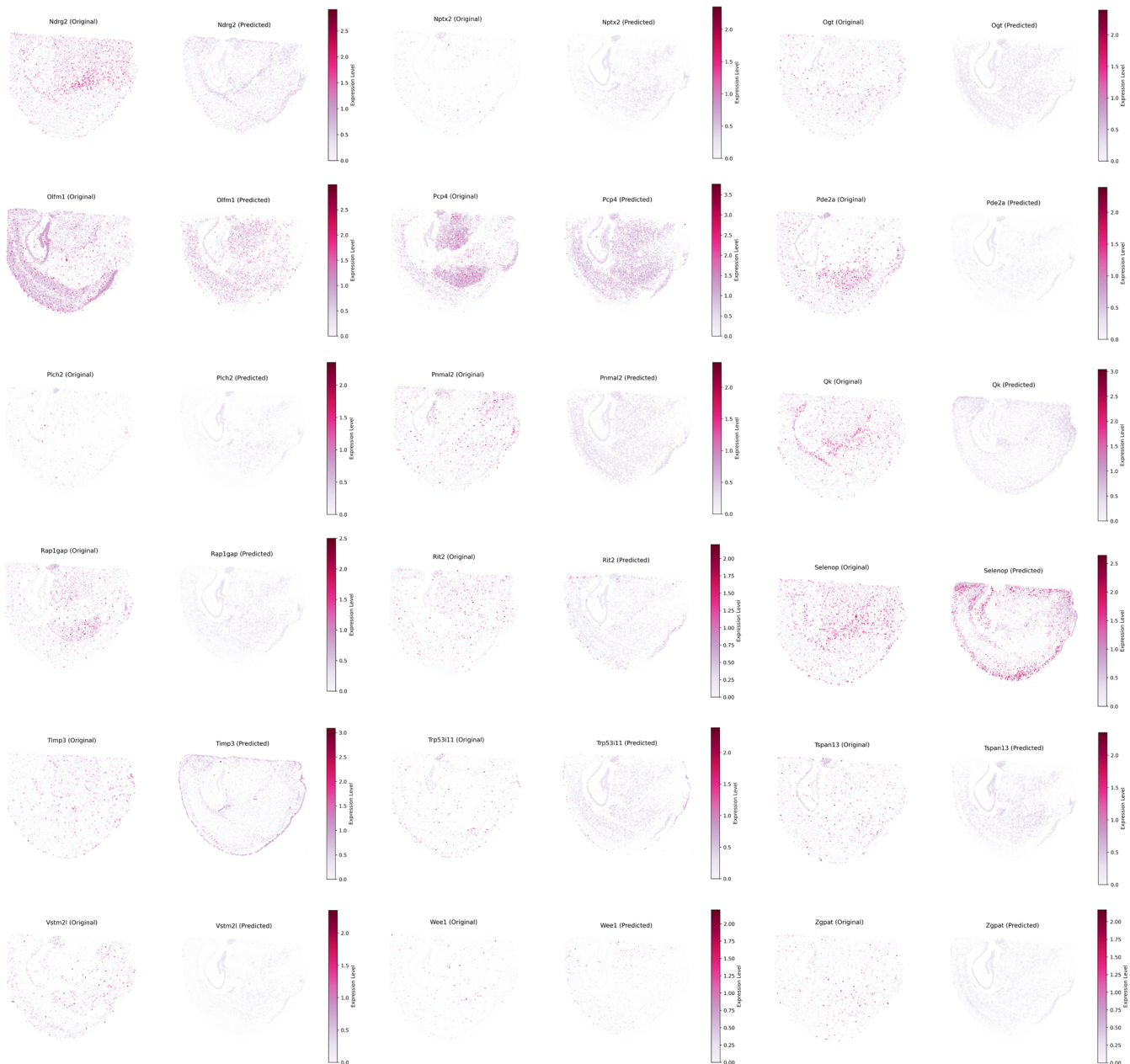

Supplement: Supplementary 1 — Figs. S1 to S9 Tables S1 to S6 [file research.0961.f1.zip › S6.pdf]

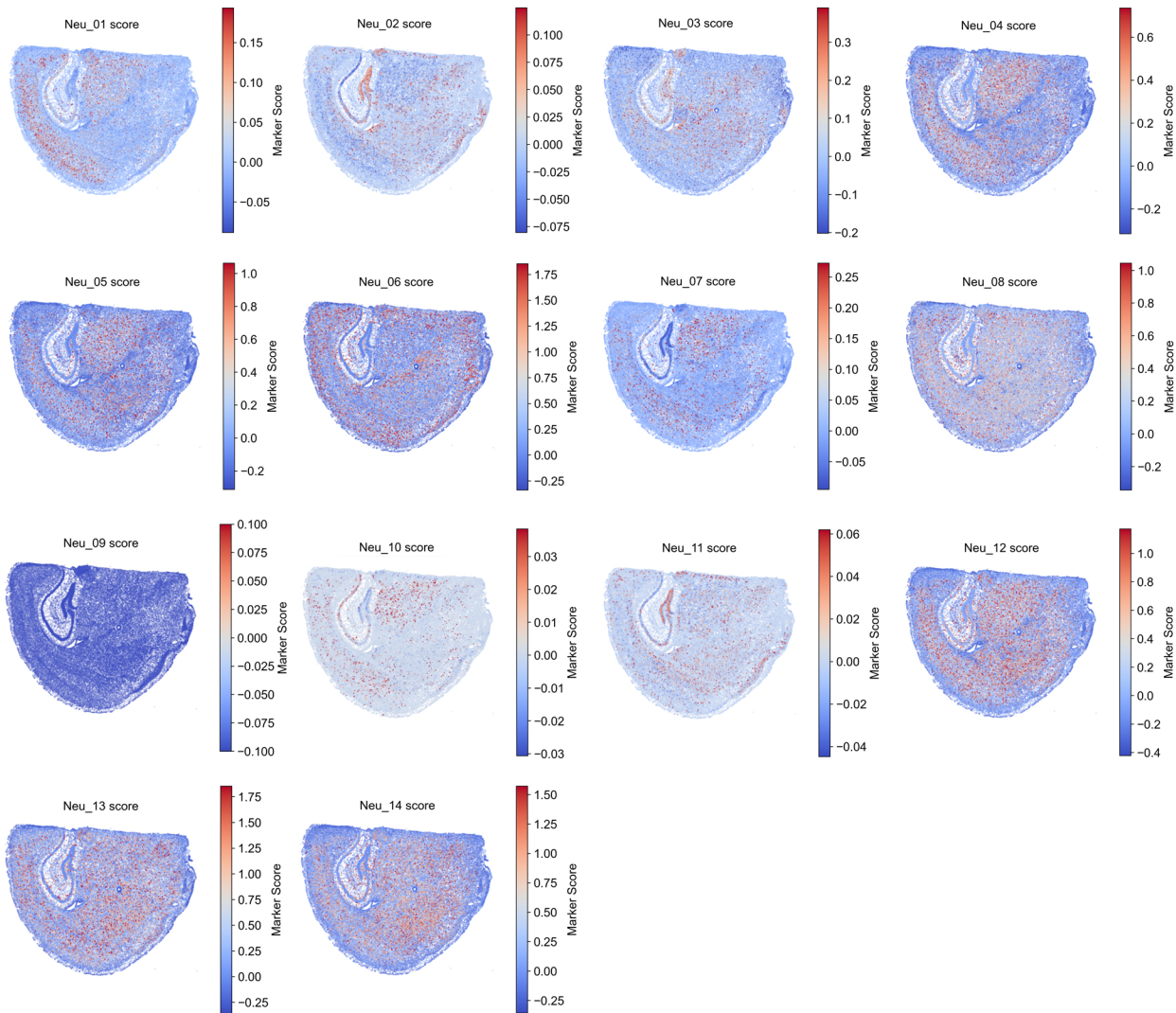

Supplement: Supplementary 1 — Figs. S1 to S9 Tables S1 to S6 [file research.0961.f1.zip › S7.pdf]

a

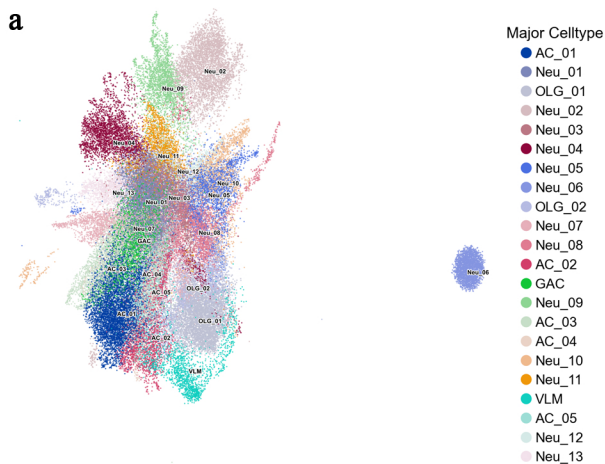

b

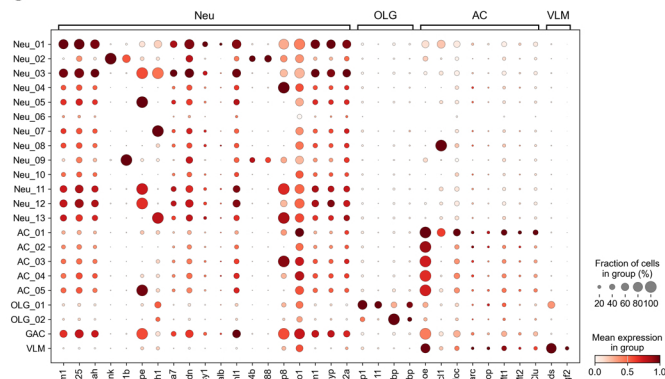

c

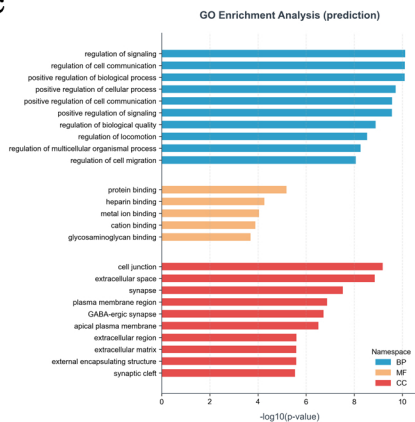

d

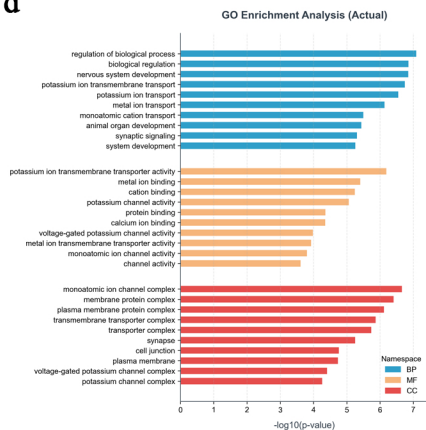

Supplement: Supplementary 1 — Figs. S1 to S9 Tables S1 to S6 [file research.0961.f1.zip › S8.pdf]

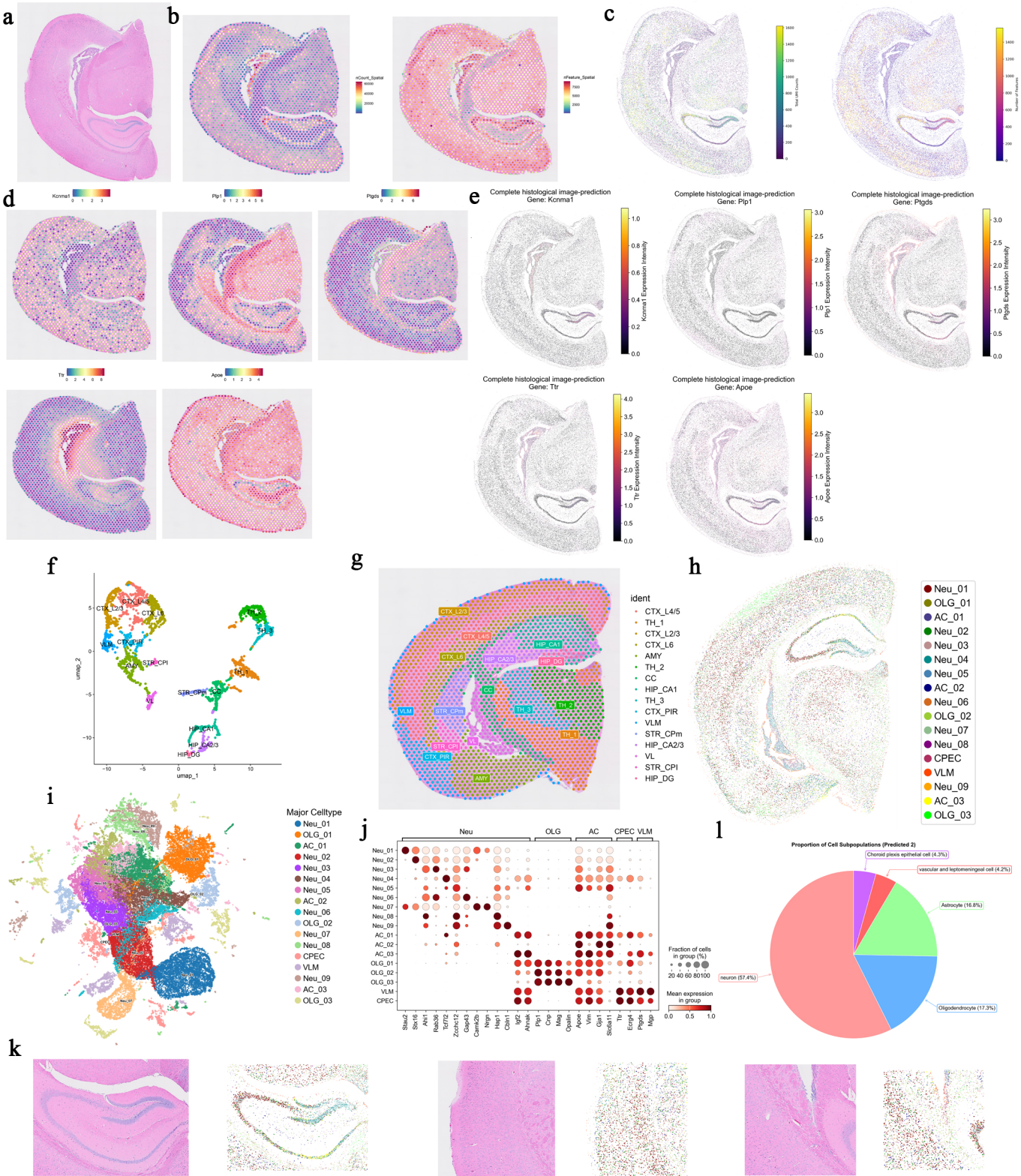

Supplement: Supplementary 1 — Figs. S1 to S9 Tables S1 to S6 [file research.0961.f1.zip › S9.pdf]
